# nanoTRON: a Picasso module for MLP-based classification of super-resolution data

**DOI:** 10.1093/bioinformatics/btaa154

**Published:** 2020-04-14

**Authors:** Alexander Auer, Maximilian T Strauss, Sebastian Strauss, Ralf Jungmann

**Affiliations:** b1 Department of Physics and Center for Nanoscience, Ludwig Maximilian University, 80539 Munich, Germany; b2 Max Planck Institute of Biochemistry, 82152 Martinsried, Germany

## Abstract

**Motivation:**

Classification of images is an essential task in higher-level analysis of biological data. By bypassing the diffraction limit of light, super-resolution microscopy opened up a new way to look at molecular details using light microscopy, producing large amounts of data with exquisite spatial detail. Statistical exploration of data usually needs initial classification, which is up to now often performed manually.

**Results:**

We introduce nanoTRON, an interactive open-source tool, which allows super-resolution data classification based on image recognition. It extends the software package Picasso with the first deep learning tool with a graphic user interface.

**Availability and implementation:**

nanoTRON is written in Python and freely available under the MIT license as a part of the software collection Picasso on GitHub (http://www.github.com/jungmannlab/picasso). All raw data can be obtained from the authors upon reasonable request.

**Contact:**

jungmann@biochem.mpg.de

**Supplementary information:**

Supplementary data are available at *Bioinformatics* online.

## 1 Introduction

**Fig. 1.**
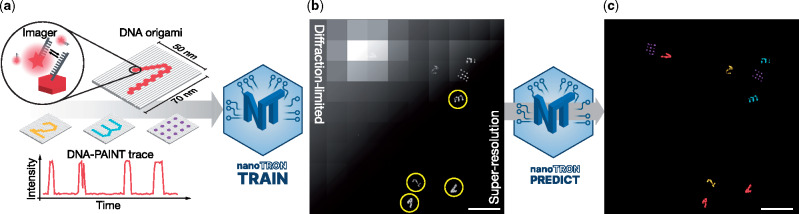
nanoTRON workflow. (**a**) DNA-PAINT imaging of DNA origami nanostructures uses transient binding of dye-labeled imager strands to their structure-bound complements, leading to target ‘blinking’ (see time trace) and enables subsequent super-resolution reconstruction of molecular patterns. (**b**) Diffraction-limited and super-resolved imaging of DNA origami structures immobilized on a glass surface. Four different patterns (Digits 1, 2, 3 and 20-nm-grid, Supplementary Fig. S1) were simultaneously imaged using DNA-PAINT. The individual patterns were grouped using Picasso’s pick function, highlighted with yellow circles. (**c**) Classified super-resolution image of the DNA-PAINT data in (b) shows the correct prediction of the four different nanoscopic patterns. Scale bars: 200 nm (b and c)

Super-resolution fluorescence microscopy allows researchers to visualize structures and dynamics below the classical diffraction limit of light (Sahl *et al.*, 2017). Stochastic super-resolution techniques use switching of fluorescent molecules between so-called dark and bright states in combination with single-molecule localization. The switching creates an apparent blinking of target molecules, which is recorded in a movie and fitted with sub-diffraction precision in post-processing, and the resulting spatial coordinates of localized fluorophores are combined into two-dimensional (2D) histograms to render a super-resolution image (Sauer and Heilemann, 2017). DNA-PAINT (Jungmann *et al.*, 2010) uses the transient binding of dye-labeled DNA oligonucleotides (called ‘imager’ strands, freely diffusing in solution) to their target-bound complementary strands (called ‘docking’ strands) to create the necessary target ‘blinking’ for super-resolution (Fig. 1). DNA-PAINT is part of a large variety of techniques, which are enabled by the use of programmable interactions of DNA molecules: DNA Nanotechnology (Ramezani and Dietz, 2019). One of the most prominent approaches in structural DNA Nanotechnology is undoubtedly DNA origami (Rothemund, 2006). Here, a long single-stranded DNA molecule is ‘folded’ via self-assembly into almost arbitrary shapes and patterns using hundreds of short oligonucleotides. DNA origami enables the manufacturing of millions of nanoscopic structures with nanometer precision in a highly controlled and parallel fashion. These very properties of DNA origami structures and their nanoscale dimensions have led to a symbiotic relationship with super-resolution approaches: DNA origami either serves as tested for assaying new super-resolution approaches (Balzarotti *et al.*, 2017; Jungmann *et al.*, 2016; Schueder *et al.*, 2019; Steinhauer *et al.*, 2009), or super-resolution is used to characterize properties of DNA nanostructures (Johnson-Buck *et al.*, 2013; Strauss *et al.*, 2018). Super-resolution instrumentation, probe design and sample preparation methods are progressing at a rapid pace, enabling cost-efficient, molecular-scale resolution on a routine basis (Auer *et al.*, 2018). Data analysis and post-processing software, however, are currently somewhat lacking behind, in most cases often still exclusively focusing on spot-detection and subsequent binning of localizations to visualize super-resolution data (Sage *et al.*, 2019). Especially more advanced yet increasingly essential post-processing tasks such as particle classification in super-resolution data is often still performed manually. Only recently, the super-resolution community turned their attention to more automated as well as machine learning- and neuronal network-based analysis approaches (Belthangady and Royer, 2019; Danial and Garcia-Saez, 2019; Ouyang *et al.*, 2018; von Chamier *et al.*, 2019). Advances in deep learning are promising for the automation of algorithmic workflows such as detecting specific shapes or pattern, e.g. recognizing handwritten digits (Lecun *et al.*, 1998). This is particularly exciting in the context of super-resolution microscopy applied to the ever-increasing complexity of DNA origami-based assays (Blanchard and Salaita, 2019). By combining super-resolution microscopy, DNA nanotechnology and deep learning, we here present a new software module, termed nanoTRON.

## 2 Implementation

nanoTRON was implemented in Python (v3.7 and higher) as a component of the Picasso software suite (Schnitzbauer *et al.*, 2017). It deploys the multi-layer perceptron (MLP) of the Python machine learning framework sci-kit learn (Pedregosa *et al.*, 2011). The software combines two of the most important workflows for model-based neural network-assisted data analysis: (i) user-friendly setup and training of artificial neural networks, (ii) classification and export of predicted data for subsequent analysis in a plug-and-play manner, see Supplementary Text S1 and S4. Super-resolution data sets can be loaded into nanoTRON for immediate classification and export, Supplementary Figure S2. The software allows the training of models for classifying of arbitrary patterns via the module ‘Train Model’ (Supplementary Fig. S2). Super-resolution data can be loaded, annotated and converted to 2D super-resolution images — gray-scale images — with a defined resolution (‘oversampling’, see Supplementary Figs S3 and S9). By rotation of every image in multiple steps, the training set can be augmented (Supplementary Fig. S3). nanoTRON supports MLPs up to three hidden layers. For the evaluation of the trained network, nanoTRON uses a train-test data split of 30% of the training set. The learning curve of the training and the confusion matrix generated from the test set visualize the performance of the trained neural network (Supplementary Fig. S2). An exemplary application with DNA origami (Supplementary Figs S1, S4–S7) is described in Supplementary Text S2. Additionally, we included a biological application with DNA origami and the nuclear pore complex (Schlichthaerle *et al.*, 2019; Thevathasan *et al.*, 2019) described in Supplementary Text S3 and visualized with Supplementary Figs S8 and S12.

## 3 Outlook

nanoTRON enables plug-and-play classification of super-resolution data using deep learning of arbitrary nanoscopic pattern. We expect nanoTRON to serve as important tool in the Picasso software collection, which due to the user-friendly design brings deep learning closer to biological researchers. We see nanoTRON as an instrument, which boosts the analysis of highly multiplexed biophysical assays, where e.g. automated detection and analysis of a plethora of barcoded structures (Lin *et al.*, 2012) for high-content and high-throughput studies would become feasible.

## Supplementary Material

btaa154_Supplementary_Data
