# Supplementary material for: nanoTRON: a Picasso module for MLP-based classification of super-resolution data
Source: Bioinformatics. 2020 Apr 14;36(11):3620–2. doi: 10.1093/bioinformatics/btaa154 (PMC7267816; doi:10.1093/bioinformatics/btaa154)
Supplement: btaa154_Supplementary_Data [file btaa154_supplementary_data.pdf]

nanoTRON: a Picasso module for MLP-based classification of super-resolution data

Alexander Auer[1,2], Maximilian T. Strauss[2], Sebastian Strauss[1,2], and Ralf Jungmann[1,2,]*

[1] Department of Physics and Center for Nanoscience, Ludwig Maximilian University, Munich, Germany. [2]Max Planck Institute of Biochemistry, Martinsried, Germany.
*Email: jungmann@biochem.mpg.de, Phone: +49 89 8578 3410

Content

**Supplementary Text 1**	Exemplary Workflow with nanoTRON
**Supplementary Text 2**	Example application with DNA origami
**Supplementary Text 3**	Example application with DNA origami and nuclear pore complexes
**Supplementary Text 4**	Recommendations and limitations of nanoTRON
**Supplementary Figure 1**	Overview DNA origami design
**Supplementary Figure 2**	Graphical user interface
**Supplementary Figure 3**	Training data generation and augmentation
**Supplementary Figure 4**	Overview of training set *Digit 1*
**Supplementary Figure 5**	Overview of training set *Digit 2*
**Supplementary Figure 6**	Overview of training set *Digit 3*
**Supplementary Figure 7**	Overview of training set *20-nm-grid*
**Supplementary Figure 8**	Overview of training set Nup96
**Supplementary Figure 9**	Exemplary rendering of one pick of the *20-nm-grid* training set
**Supplementary Figure 10**	Model parameter tuning of the numbers of nodes in the 1-layer network
**Supplementary Figure 11**	Training and test score with different training set sizes using the 1-layer network
**Supplementary Figure 12**	Proof-of-concept experiment with biological target
**Supplementary Table 1**	Experimental conditions training set *Digit 1*
**Supplementary Table 2**	Experimental conditions in training set *Digit 2*
**Supplementary Table 3**	Experimental conditions training set *Digit 3*
**Supplementary Table 4**	Experimental conditions in training set *20-nm-grid*
**Supplementary Table 5**	Experimental conditions in validation set
**Supplementary Table 6**	M13mp18 p7249 sequence
**Supplementary Table 7**	Rectangular DNA origami staple strands
**Supplementary Table 8**	Biotinylated staple strands
**Supplementary Table 9**	DNA-PAINT docking site sequences

**Supplementary Table 10**	DNA-PAINT imager sequences
**Supplementary Table 11**	Training runtime comparison with various computers
**Supplementary Table 12**	Prediction runtime comparison with various computers
**Supplementary Table 13**	nanoTRON MLP compared with LeNet-5 CNN
**Supplementary Table 14**	LeNet-5 CNN Design
**Materials and buffers**	
**Optical setups**	
**DNA origami self-assembly**	
**Nanobody conjugation**	
**Super-resolution DNA-PAINT imaging with DNA origami**	
**Super-resolution DNA-PAINT imaging with nuclear pore complex**	
**Super-resolution reconstruction**	
**References**	

Supplementary Text 1 | Exemplary Workflow with nanoTRON

nanoTRON Train

(1) **Collecting training data:** Training data can be either generated with dedicated experiments for every class, or already existing data for the nanopatterns can be utilized. In any case, training data for every class should be gathered.

Tipp: Picasso command-line tool Picasso *csv2hdf* allows the conversion from ThunderSTORM .csv localization tables to the Picasso format.

(2) **Selecting nanopattern:** After spot identification and localization using Picasso *Localize* was performed, one can visualize and, if necessary, drift correct the localization files in Picasso *Render*. Using the *Pick Tool* the nanopattern can be selected manually. Another function called *Pick Similar* provides an automated solution for picking patterns in the whole field of view. Therefore, one selects a few nanopatterns manually by hand and applies *Pick Similar*. It utilizes the predictable blinking kinetics of DNA-PAINT and selects regions with similar number of localizations in areas of the size of the pick diameter. Every pick gets assigned with a group id, see **Supplementary Figure 3a**. The picked localizations can be saved using *File → Save picked localizations*.

(3) **Setting up nanoTRON Train:** If training data for every picked nanopattern is available, the training files can be loaded into the module nanoTRON *Train*, see **Supplementary Figure 2a***.* First, the number of unique patterns needs to be set. In the box *Training Files,* all the files can be loaded and assigned with a class name. If necessary, the oversampling parameter can be modified, see **Supplementary Figure 3b**. *Expand Training Set* can be enabled to leverage the training data by augmentation, see **Supplementary Figure 3c**. After the image parameters are set up, *Prepare Data* converts the localization tables into grayscale images, see **Supplementary Figure 3b** and **Supplementary Figure 9**. In the box *Perceptron,* the neural network can be tuned. See the exemplary application described in **Supplementary Text 2** for more details on this step.

Attention: nanoTRON Train does not allow for duplicated class names. Every class needs to be assigned with a unique class name for the model.

Tipp: With *Export Image Subset* ten images of every class can be exported. They are saved in the training file path.

(4) **Training:** After the perceptron is set up accordingly, the training can be started with the button *Train*. The runtime of training can take up to hours, see **Supplementary Table 11** for a comparison between different hardware configurations. When the training has finished, the learning curve and confusion matrix can be inspected with *Show Learning Curve*. Using *Save Model,* the trained neural network can be saved for later use.

nanoTRON Predict

(1) **Collecting target data:** After the target data is processed with Picasso *Localize*, the nanopatterns are selected in Picasso Render using the Pick Tool, as described in the section Selecting nanopattern for training.

(2) **Prediction:** The grouped localization file can be loaded into nanoTRON predict via drag and drop or *File → Open.* The corresponding model can be imported via *Tools → Load model.* All available classes for prediction are listed in the box *Export Structures* The prediction is started with the button *Predict*.

(3) **Export:** After the prediction finished, the classified nanopatterns can be exported in separate files. All nanopatterns, which should be exported, can be selected in the box *Export Structures*. Finally, nanoTRON exports all selected nanopatterns using the button *Export*.

Tipp: With *Filter Probabilities,* the classified nanopattern can be filtered according to the prediction score.

Tipp: With *Export Pick Regions,* a table of pick regions can be exported additionally to the localization tables.

Attention: The option *Regroup Export Files* reassignes the picks with new group ids for every exported file. The group ids before prediction do not correspond to the reassigned group ids.

Supplementary Text 2 | Example application with DNA origami.

As a proof-of-concept demonstration, we acquired five DNA-PAINT (Jungmann, et al., 2010) super-resolution example data sets, each containing DNA origami (Rothemund, 2006). Four data sets display a unique DNA origami pattern of digits 1 to 3 or a 3×4-grid-structure with 20-nm-spacing, **Figure 1a** and **Supplementary Figure 4-7**. A subsequent acquisition with all four DNA origami designs in a single sample serves as a validation data set, **Figure 1b**. Imaging conditions are described in **Supplementary Table 1-5**, DNA origami design sequences are listed in **Supplementary Table 6-10**.

Using a 1-hidden-layer perceptron with 550 nodes and *ReLU* (Hahnloser, et al., 2000; He, et al., 2015) activation function and *adam* solver (Kingma, 2014), we could achieve a training accuracy of ~ 99%, test accuracy of ~ 98% and a validation accuracy ~ 94%, **Figure 1c** and **Supplementary Figure 10**. In the validation set, unidentifiable structures caused e.g. by structure misfolding, clustering, or loose attachment to the surface, were manually selected and excluded from the validation.

Supplementary Text 3 | Example application with DNA origami and nuclear pore complexes.

As a proof-of-concept demonstration for the applicability with biological samples, we generated an artificially merged DNA-PAINT super-resolution data set, displayed in **Supplementary Figure 12**. It contains the validation data set with the DNA origami structures (digits 1-3 and the 3×4-grid-structure with 20-nm-spacing) of **Figure 1c** and biological DNA-PAINT super-resolution data of the GFP-tagged nuclear pore complex (NPC) protein Nup96. The artificial data set was generated in the following way, that a mask of the NUP96 related area of a 512 × 512 px super-resolution image the NUP96 experiment was created using Picasso: *Mask*, available in Picasso *Render*. The mask was then applied to the 512 × 512 px DNA origami validation image so that the Nup96 related areas were cleared of DNA origami localizations. Using the command-line function *picasso join file1 file2* the Nup96 localization file and the masked DNA origami localization file were combined. The artificial localization file was then loaded into Picasso *Render* and a few nuclear pore complexes and DNA origami were selected manually with the *Pick Tool*. Afterward, the whole image was screened for nuclear pore complexes and DNA origami with the automation picking tool *Pick Similar,* resulting in 12681 picks.

For the classification of the DNA origami and NPCs, we used the four training sets of the DNA origami, **Supplementary Figures 4-7**, and one additional DNA-PAINT recording of the NUP96 labeled nuclear pore complex shown in **Supplementary Figure 8**. The trained model for the five classes achieved 99% training and 98% test accuracy. The neural network design was used as described in **Supplementary Text 2**. Oversampling was set to 40 and pick diameter to 1.5 px, resulting in grayscale images of 60 × 60 px size.

Supplementary Text 4 | Recommendations and limitations of nanoTRON.

To make nanoTRON useful as a standard tool in data analysis, we here provide a few recommendations for best practices. Successful classification strongly depends on the quality of the training and the training data (Belthangady and Royer, 2019). Like every deep learning framework, nanoTRON has limitations in performance and usage. To best prepare the user, we want to comment on a few limitations and mitigation approaches.

Recommendations

Training data size: The training set should contain a sufficient number of picks in every class. We recommend at least 200 picks per class, see **Supplementary Figure 11**. If possible, higher number of picks per class is favourable.

Balanced data sets: The whole training set should be balanced, meaning that the number of picks in every class should be similar. Unbalanced training sets can cause training and prediction artefacts.

Data set augmentation: For training, we always recommend the data augmentation option *Expand training set*. Increasing the number of training data by rotations yields higher training and test accuracy, see **Supplementary Figure 11.**

Neural network design: For the classification of nanopatterns similar to the examples in **Supplementary Text 1** and **Supplementary Text 2**, we propose to use a comparable layer design: 1 layer with 550 nodes and ReLU as activation function, see **Supplementary Figure 10** for more details.

Hyper parameter testing: We recommend testing different configurations for hyperparameters, like the number of layer and nodes, activation function etc. for training to achieve the best performing model.

Image configuration: The parameter oversampling depends on the resolution of the super-resolution data. In combination with the pick diameter, an image input size of 40–60 px should be ideal. We suggest using lower oversampling as the resolution of the super-resolution data would provide.

Validation experiments: We want to stress that new models should not be trusted “blindly”. Validation experiments should be made to understand the applicability and limitations of the trained model.

Limitations

Computation time: In principle, there is no limitation in the size of the nanopatterns. However, increasing the size (pick diameter) with constant oversampling will also increase the image size and therefore computation time. Runtimes of training can last up to hours and days for very large nanopatterns.

Computation resources: We recommend ≥16GB RAM for training with nanoTRON.

Discovery: nanoTRON will not discover new nanopatterns in the prediction data set. Structures, which were not included in training will be incorrectly classified. Therefore, for every unique nanopattern one needs to prepare training data and include that into the model.

Model size: nanoTRON Train GUI is limited to 10 different classes.

Data quality: The data quality of the training data set but also prediction data set strongly influences the performance of nanoTRON. Low quality data will likewise result in poor performance.

Reproducibility: nanoTRON model system is designed to export the model file in .sav format along with an YAML documentation file, which contains all necessary parameters of the trained model. Values for hyperparameters, as well as the path to the training files. While train and test accuracies are included in the documentation file, we propose saving also the learning curve and confusion matrix after training.

Generalization: Neural network training can suffer from “overfitting”, i.e. that a model performs well on the training data but fails to generalize on new data. In the context of super-resolution microscopy data, this could happen when the resolution of training and new data is different. Therefore, we recommend combining

multiple super-resolution images of the same class with varying spatial resolution for the training set, as suggested by Belthangady and Royer. The command-line function *picasso join file1 file2* offers a tool for combining localization files, see **Supplementary Text 3**. Combining multiple files will train the model for a more general usage. Attention: Picking the nanopatterns needs to be done after combining, otherwise the group ids will be doubled.

Artefacts: Real-world experiments contain artefacts and background signal. In the case of DNA origami, this could e.g. be misfolded structures. With biological targets, labelling issues can generate unwanted background signals. While selecting the nanopatterns with Picasso *Render* - especially if *Pick Similar* is used – we recommend screening the picks for artefacts and interactively excluding them in the training and prediction data set.

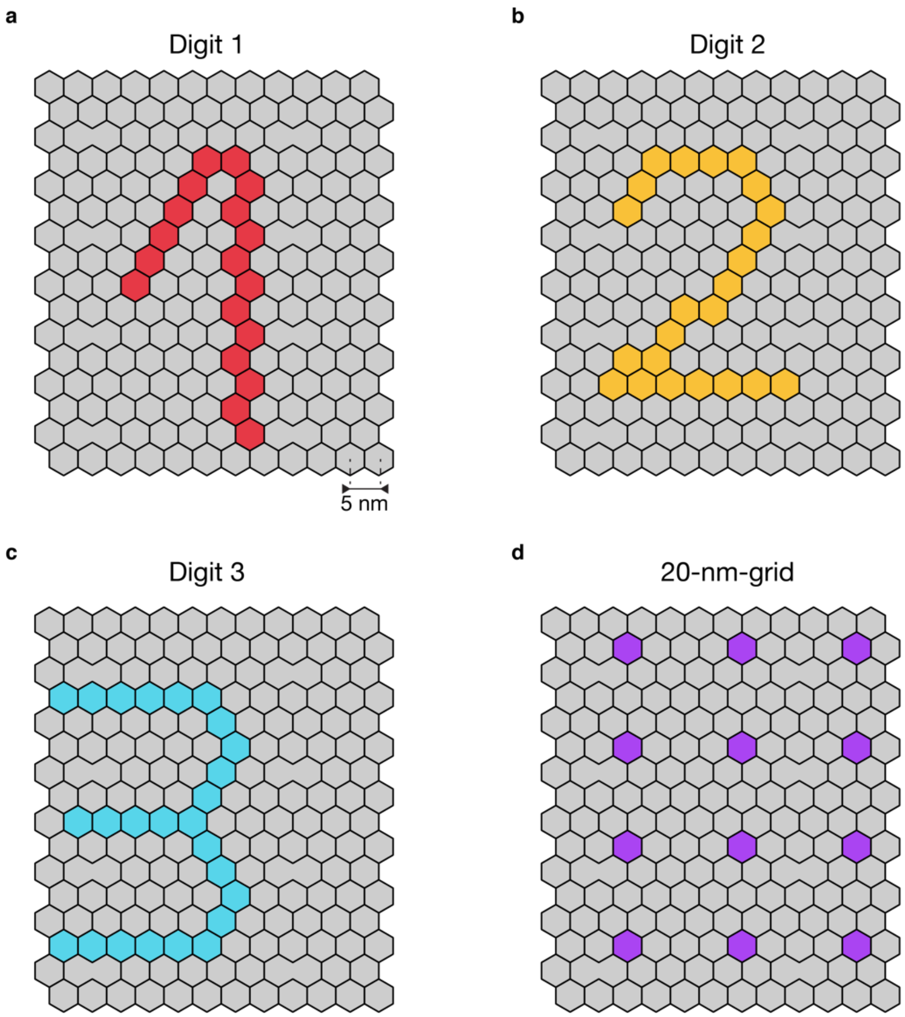

Supplementary Figure 1 | Overview DNA origami design. (**a**) Design of the ‘Digit 1’ structure. Red labeled hexagons mark the DNA staples, which are extended with the P1 docking sequence (**Supplementary Table 10**) for DNA-PAINT super-resolution imaging. Hexagon-to-hexagon distance is ~ 5 nm. (**b**) Design of the ‘Digit 2’ DNA origami. Yellow hexagons indicate the P3 DNA-PAINT docking sites. **c**) Design of the ‘Digit 3’ DNA origami. Cyan hexagons mark the P5 DNA-PAINT docking sites. **d**) Design of the ’20-nm-grid’ DNA origami, a 3×4-grid-structure with 20 nm spacing. Hexagons colored magenta identify the P1 DNA-PAINT docking sites.

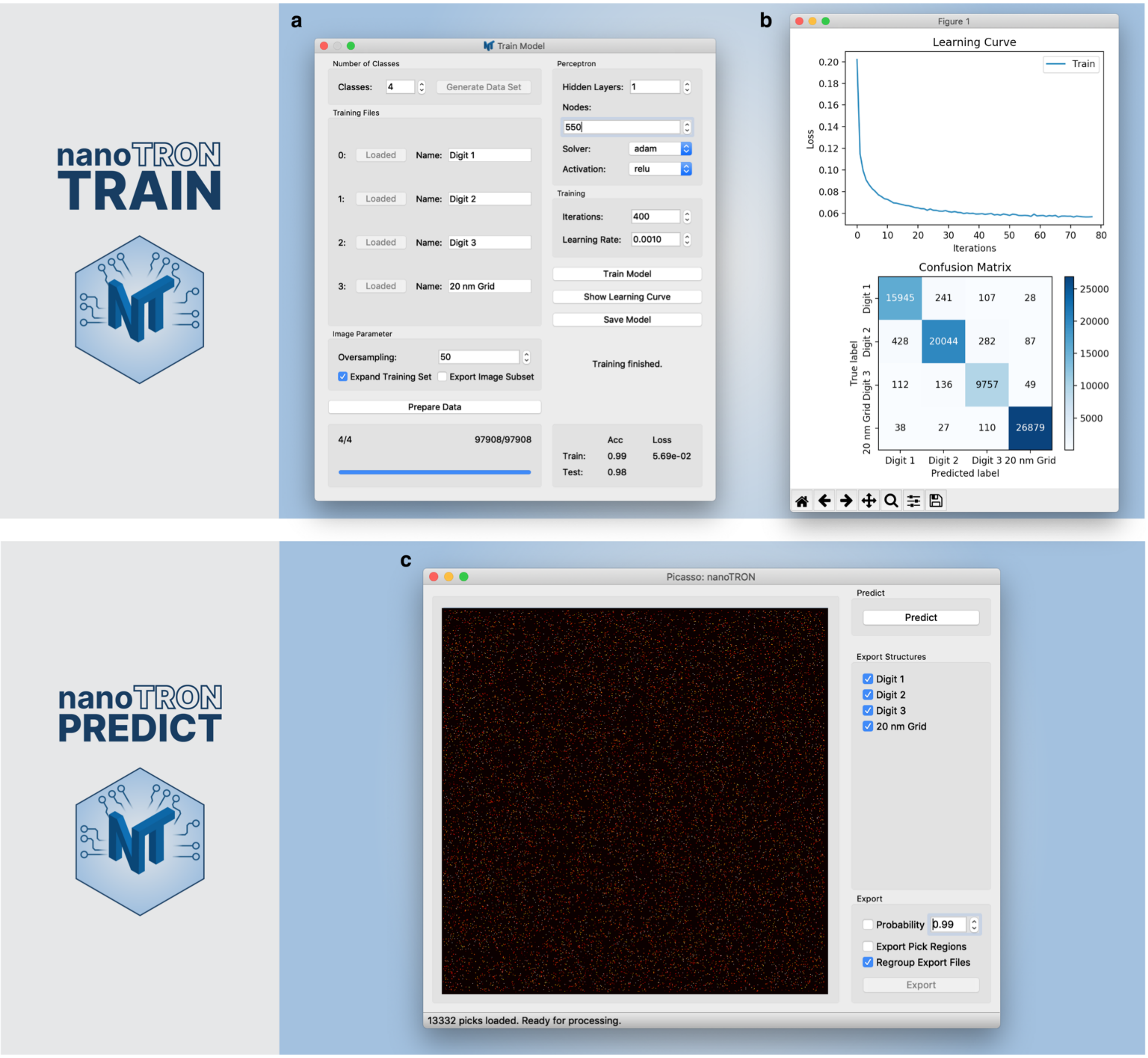

Supplementary Figure 2 | Graphical user interface. (**a**) GUI of nanoTRON: Train. Super-resolution training data sets are loaded into nanoTRON and converted to pixel images (**Supplementary Figure 3** and **Supplementary Figure 9**). The artificial neural network is set up, trained, and saved. (**b**) Performance of the network can be visualized with a plot of the learning curve and the confusion matrix. (**c**) GUI of nanoTRON main window. Super-resolution data can be loaded into nanoTRON via drag and drop. Either a default or a saved model (Tools → Load model) of the artificial neural network can be used to classify the nanopatterns in the super-resolution data. The default model gets loaded when the software is started. After prediction, the labeled data can be filtered using the predicted probability and exported as individual data sets with the corresponding meta data file (YAML file). In addition to the super-resolution data, the Picasso's *pick regions* can be exported and subsequently used for further analysis.

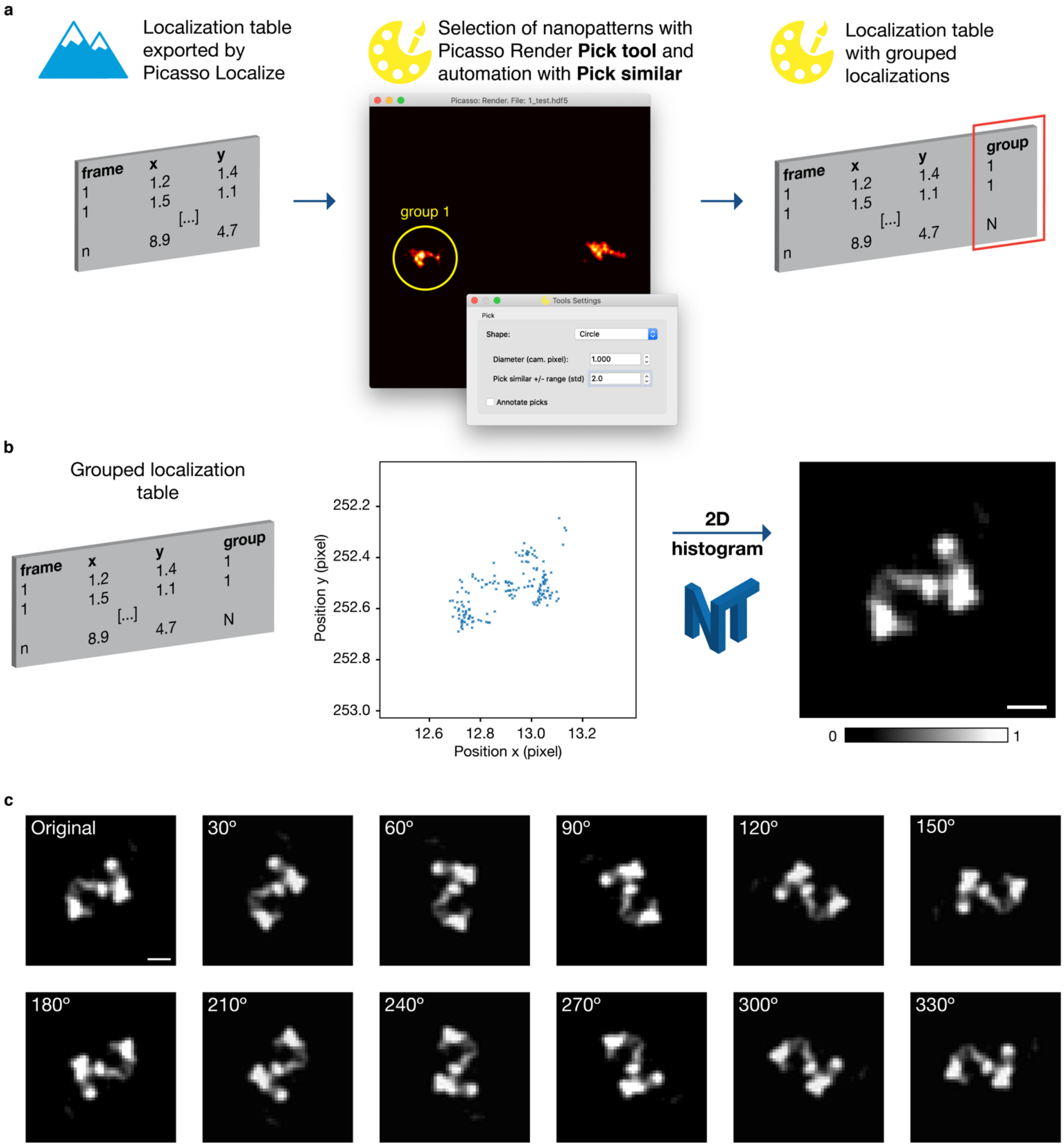

Supplementary Figure 3 | Training data generation and augmentation. (**a**) In localization-based super-resolution microcopy, diffraction-limited images get "converted" into tables of localizations by estimating the centers of single molecule emissions. In Picasso, the module *Localize* provides the graphical user interface for processing raw microscopy data and turning them into localization tables. In Picasso *Render,* the localization tables can then be rendered as an image. To utilize nanoTRON, first nanopatterns need to be selected. Using Picassos *Pick Tool,* nanopatterns can be manually selected by a center point and a pick diameter. One super-resolution image of e.g. DNA origami with 512 x 512 px can contain up to tens of thousands of nanopatterns. The tool *Pick similar* provides an automated solution for screening the whole image and picking comparable areas. Every pick is then assigned with a unique group id. (**b**) During training data preparation in nanoTRON, the localizations are converted into grayscale images and normalized between 0 and 1. Every pick corresponds to one nanopattern and consequently one grayscale image. One exemplary heatmap of a *20-nm-grid* pick is visualized in **Supplementary Figure 9**. The resolution of the image can be set via the parameter 'oversampling', **Supplementary Figure 2a**. (**c**) The training set data can be augmented with rotated variants of every image. Ultimately,

the original rendering of the super-resolution data is rotated 11 times around the center-of-mass with a step size of 30º effectively increasing the training data 11-fold. Scale bars, 20 nm (**a**, **b**)

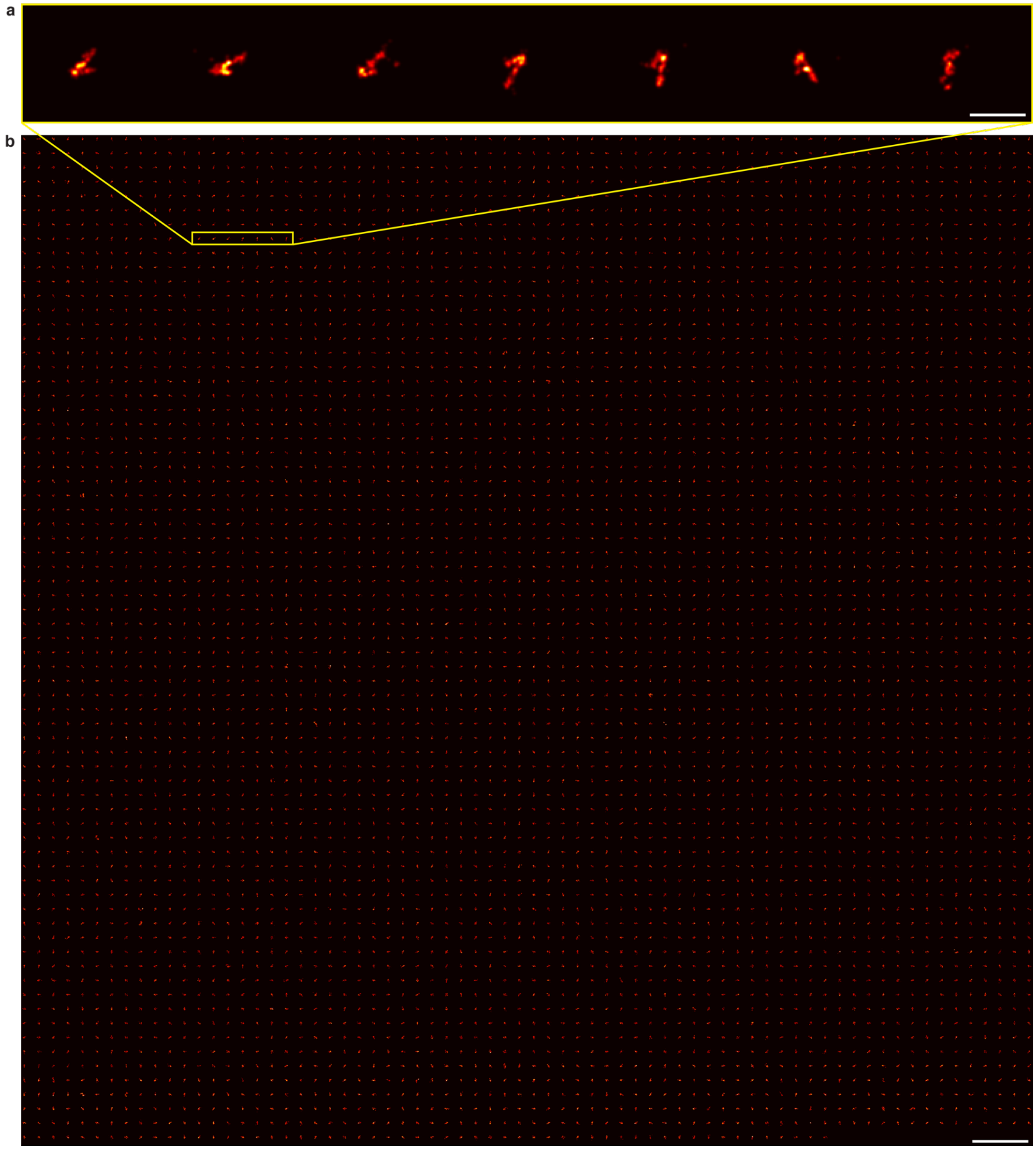

Supplementary Figure 4 | Overview of training set *Digit 1*. (**a**) Zoom-in of individual DNA origami imaged with DNA-PAINT (**b**) DNA-PAINT super-resolution mosaic image of 4955 DNA origami patterned with digit 1 (shown in **Supplementary Figure 1a**) DNA-PAINT docking sites with Sequence P1. Scale bars, 100 nm (**a**), 1 μm (**b**).

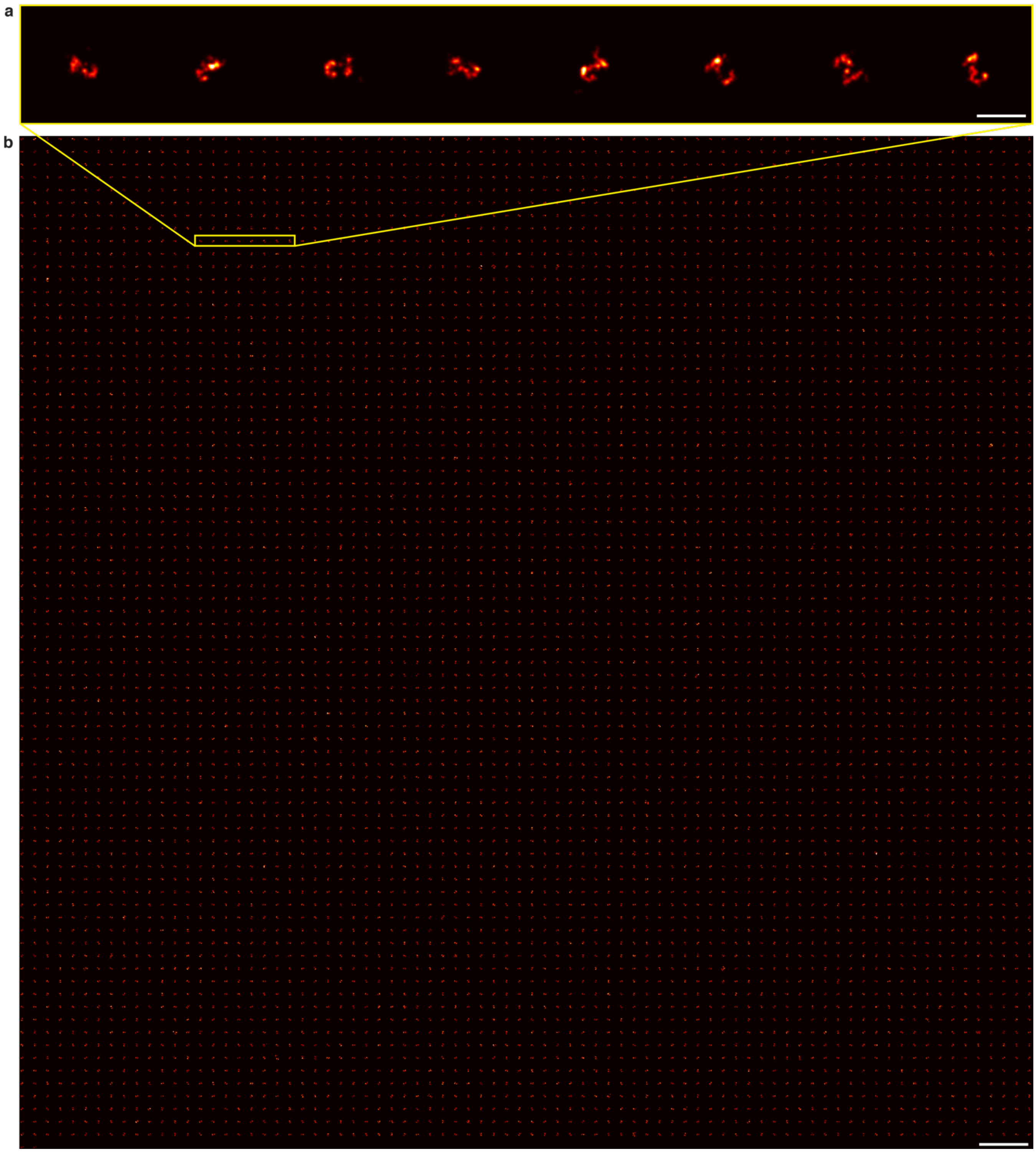

Supplementary Figure 5 | Overview of training set *Digit 2*. (**a**) Zoom-in of individual DNA origami imaged with DNA-PAINT (**b**) DNA-PAINT super-resolution mosaic image of 6321 DNA origami patterned with digit 2 (shown in **Supplementary Figure 1b**) DNA-PAINT docking sites with Sequence P1. Scale bars, 100 nm (**a**), 1 μm (**b**).

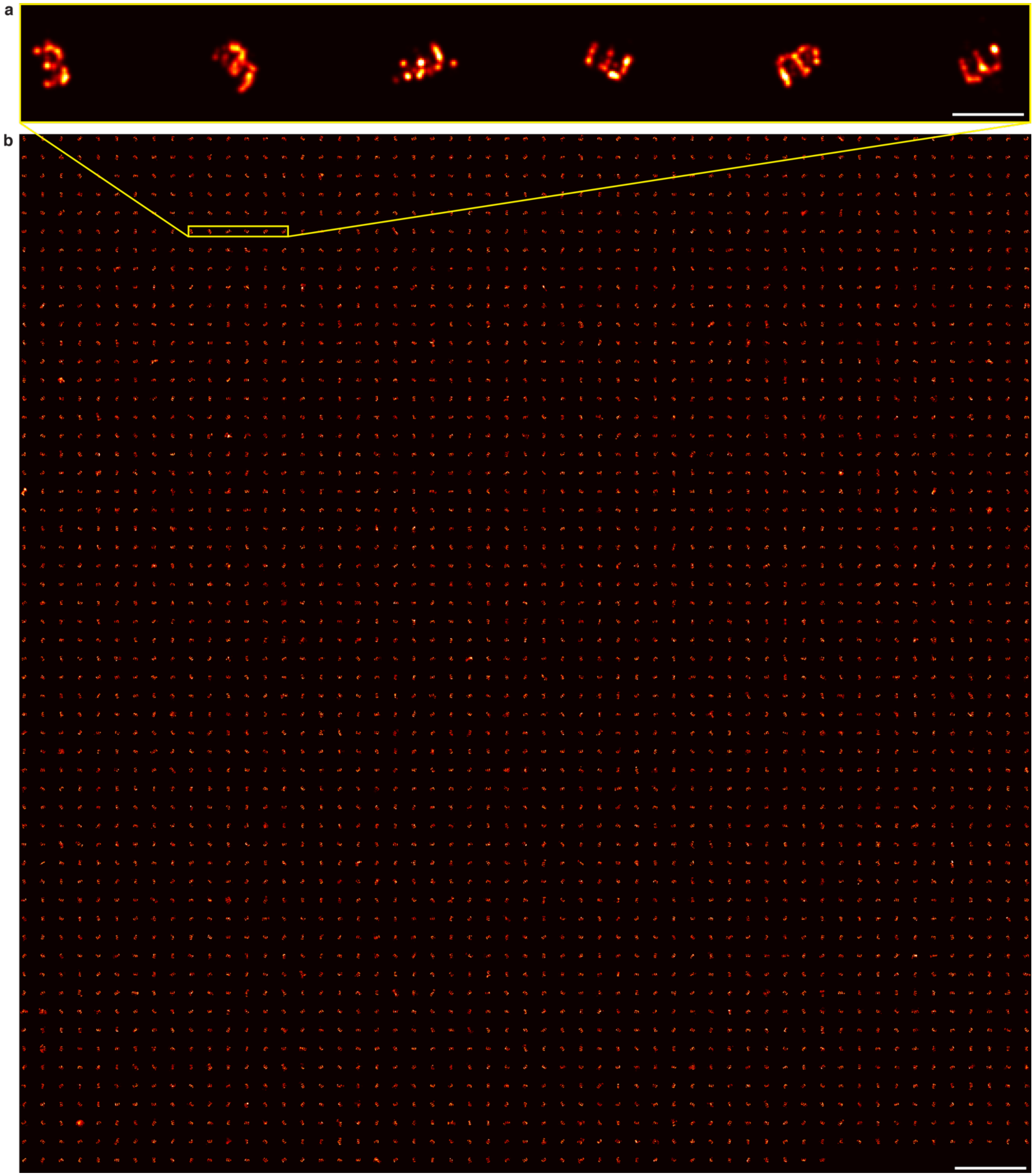

Supplementary Figure 6 | Overview of training set *Digit 3*. (**a**) Zoom-in of individual DNA origami imaged with DNA-PAINT (**b**) DNA-PAINT super-resolution mosaic image of 3068 DNA origami patterned with digit 3 (shown in **Supplementary Figure 1c**) DNA-PAINT docking sites with Sequence P1. Scale bars, 100 nm (**a**), 1 µm (**b**).

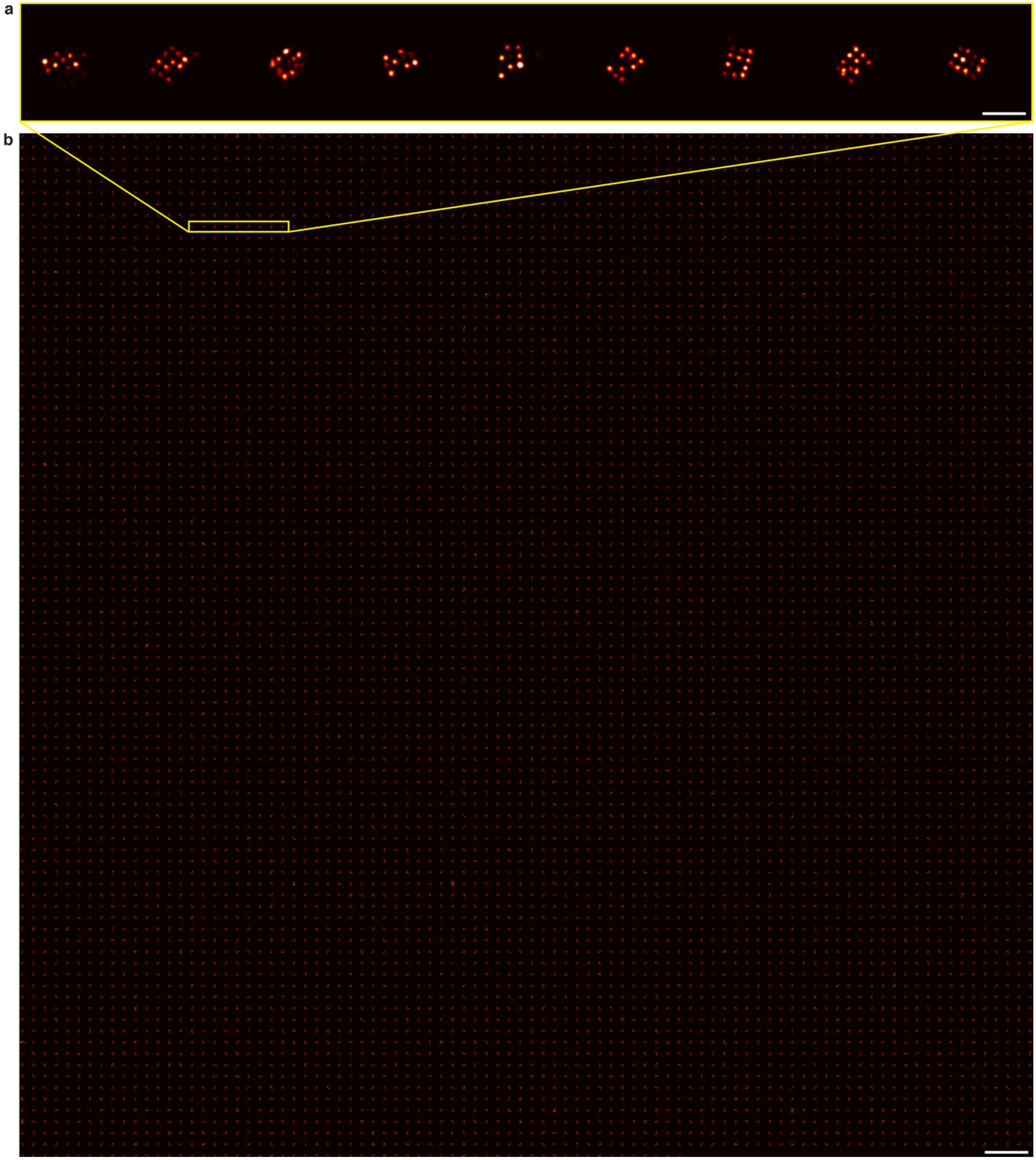

Supplementary Figure 7 | Overview of training set *20-nm-grid*. (**a**) Zoom-in of individual DNA origami imaged with DNA-PAINT (**b**) DNA-PAINT super-resolution mosaic image of 6321 DNA origami patterned with a 3 × 4 grid with 20 nm spacing (shown in **Supplementary Figure 1d**) DNA-PAINT docking sites with Sequence P1. Scale bars, 100 nm (**a**), 1 µm (**b**).

a

b

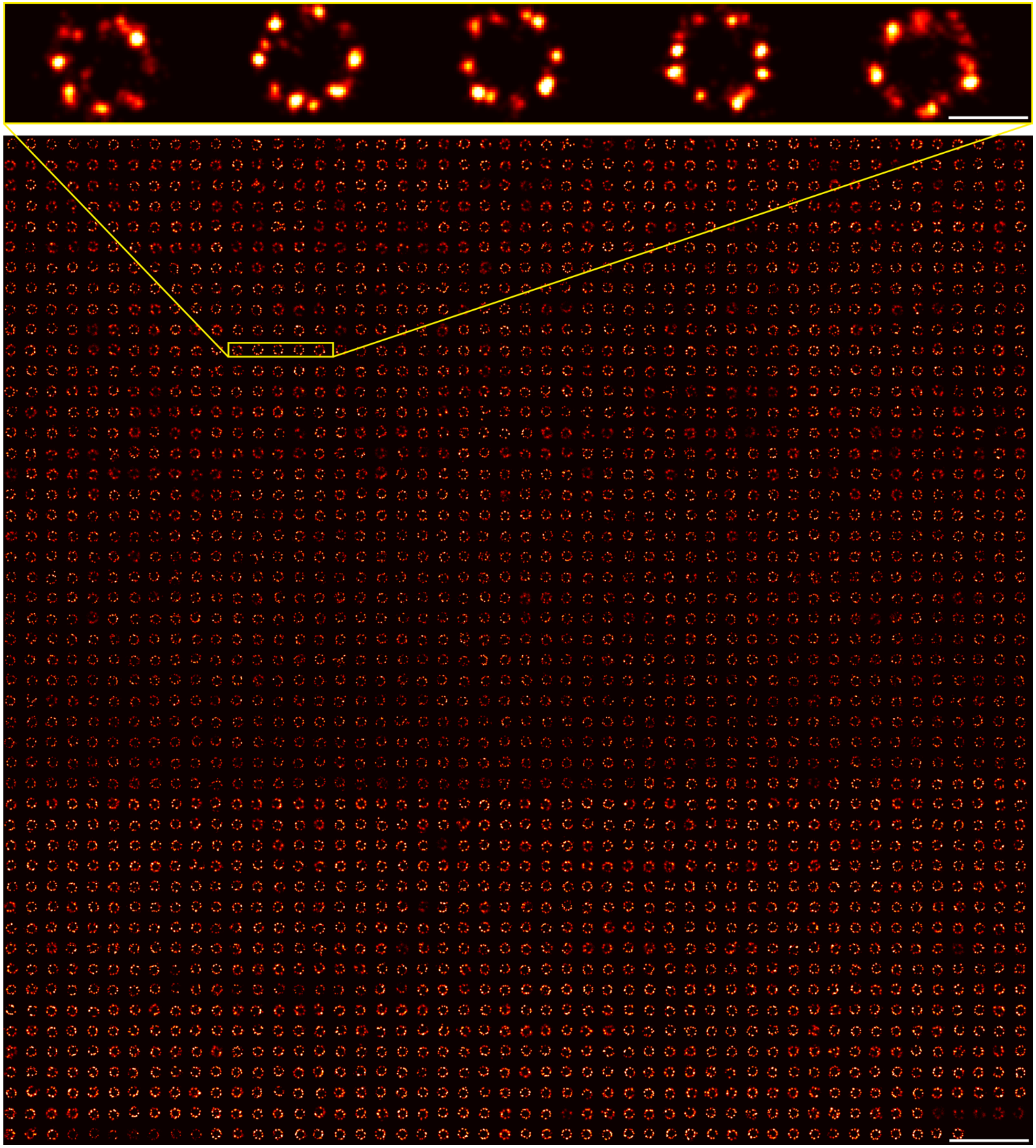

Supplementary Figure 8 | Overview of training set Nup96. (**a**) Zoom-in of individual Nup96 proteins of the nuclear pore complex in a fixed U2OS cell. (**b**) DNA-PAINT super-resolution mosaic image of 2447 nuclear pore complexes labeled with DNA-modified GFP nanobody. Scale bars, 100 nm (**a**), 1 µm (**b**).

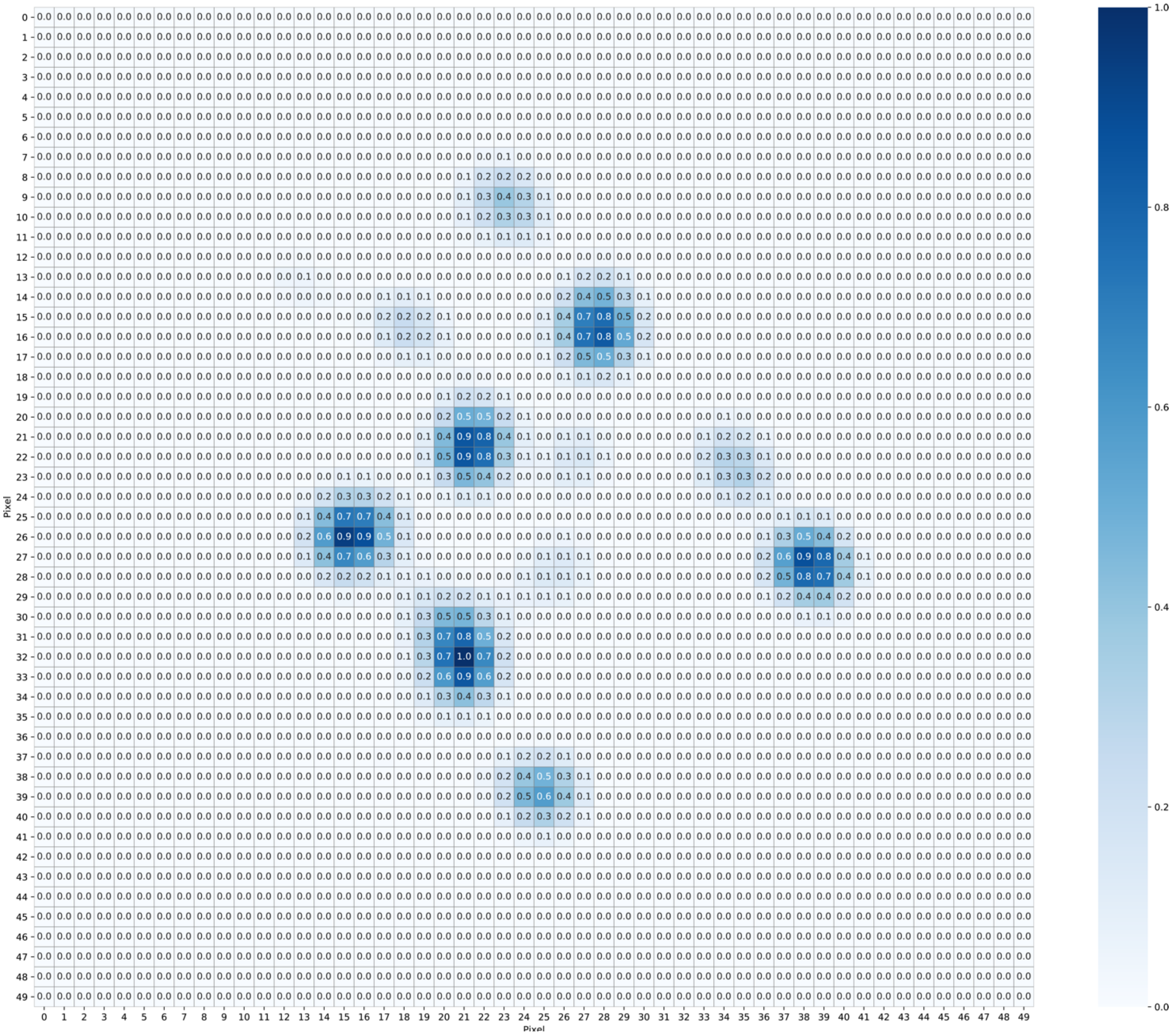

Supplementary Figure 9 | Exemplary heatmap of one pick of the *20-nm-grid* training set. While preparing the data for training, nanoTRON converts the localizations of picks into grayscale images, as illustrated in **Supplementary Figure 3b.** The size of the image corresponds to the pick diameter and the chosen oversampling according to image size = pick diameter × oversampling. Every image gets scaled to gray values from 0 to 1. After converting all training sets to image stacks, the MLP is trained with the grayscale images. The exemplary heatmap displays one *20-nm-grid* pick after conversion from localizations to an image with rounded gray values for clear visualization. nanoTRON does not round the gray values.

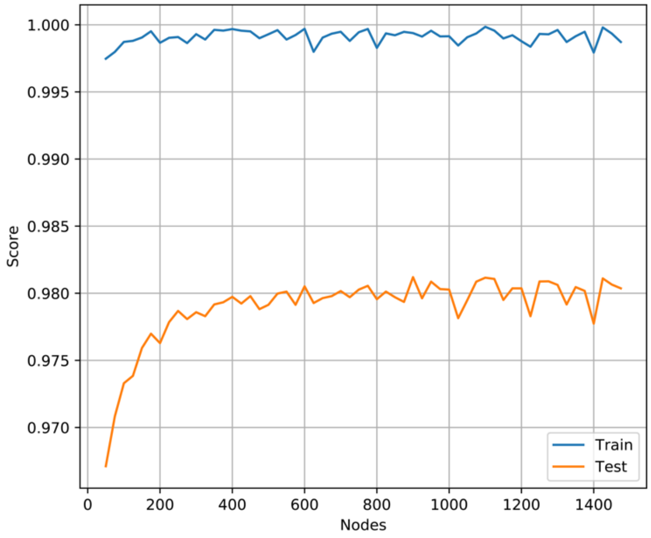

Supplementary Figure 10 | Model parameter tuning of the numbers of nodes in the 1-layer network. Training and test score achieved with the four classes training set with varying number of nodes from 50 to 1500. The final value was set to 550 nodes, indicating sufficient model complexity. Further increasing the number of nodes did not increase the test accuracy.

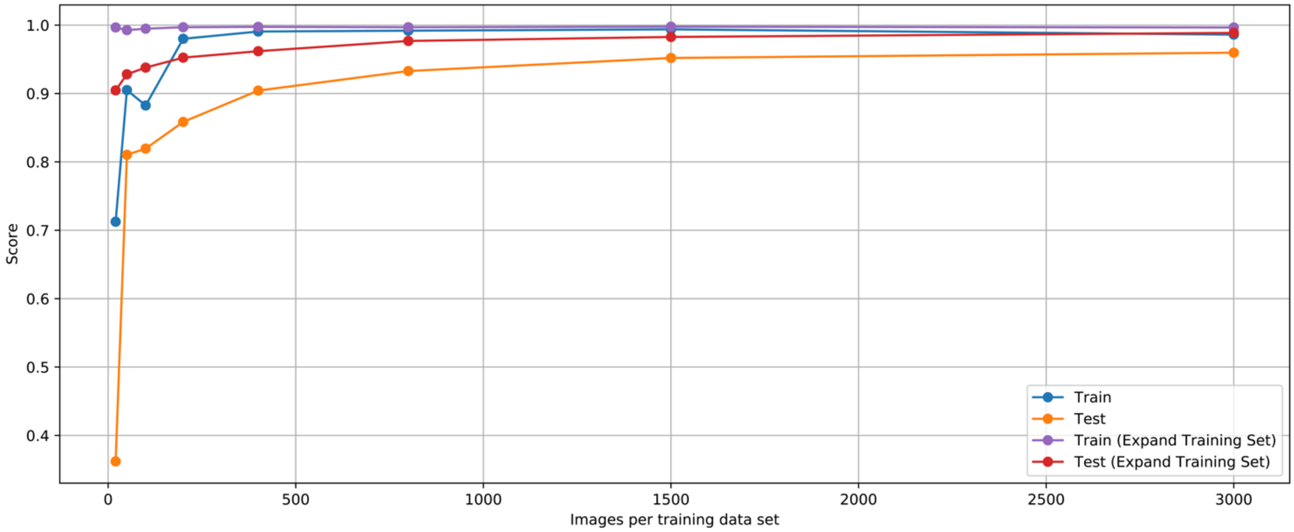

Supplementary Figure 11 | Training and test score with different training set sizes using the 1-layer network. Training and test scores achieved using the 4 classes. The number of picks in every unique training set were varied, starting from 20 up to 3000 picks per set. The scores were calculated with and without the nanoTRON option “Expand Training Set”, **Supplementary Figure 3c**. Using 200 images per unique training set and the data augmentation option, a test accuracy of ~0.95 could be realized. Without augmentation the test accuracy dropped to ~0.86. Larger training sets with 3000 picks per unique set increase test accuracy up to almost ~0.99.

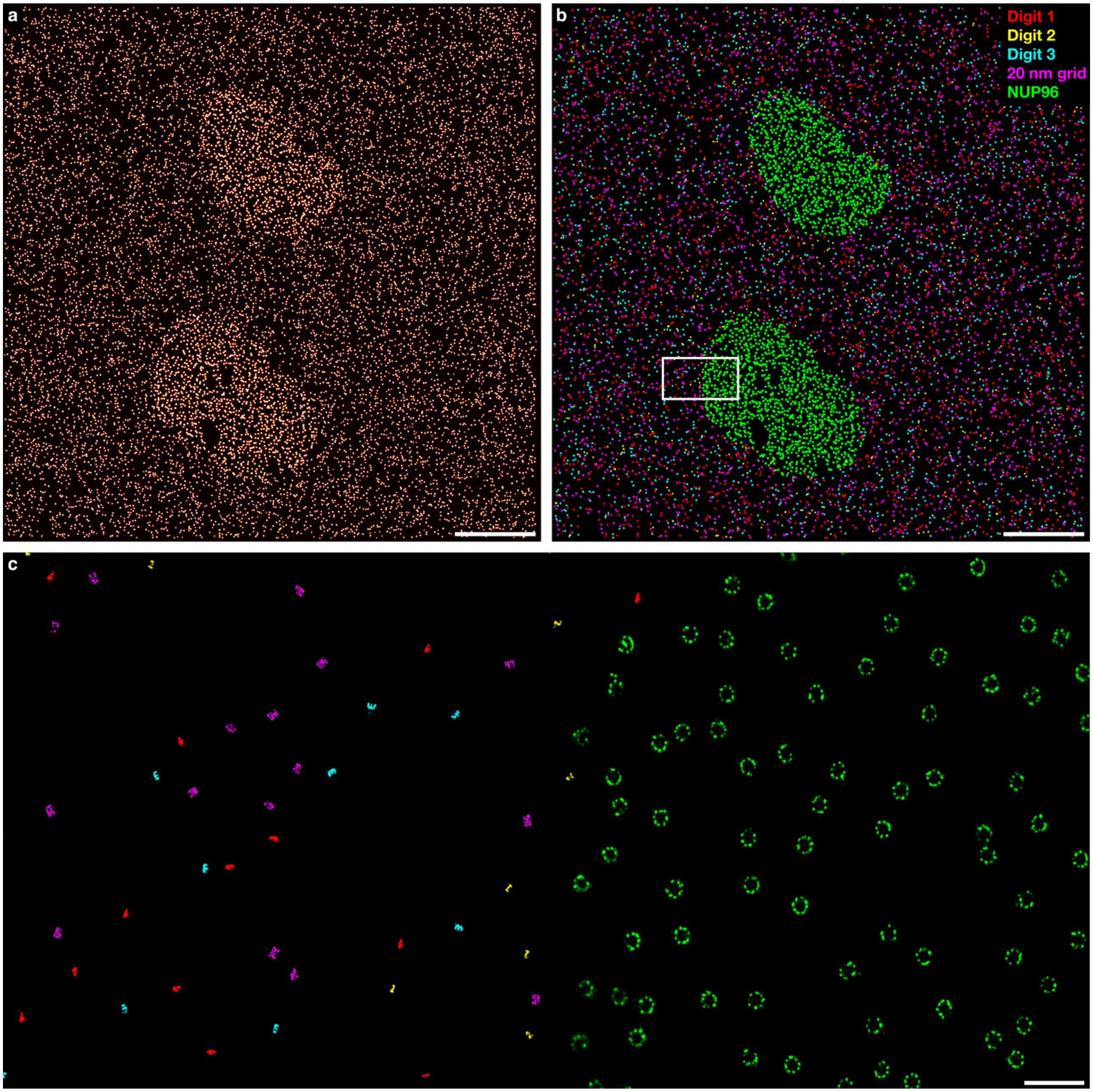

Supplementary Figure 12 | Proof-of-concept experiment with a biological target. (**a**) Overview image of the artificial DNA-PAINT data set constructed as described in **Supplementary Text 3**. (**b**) Super-resolution image with classified nanopatterns using nanoTRON and a 5-class model, which was trained as described in **Supplementary Text 3**. The different colors (red, yellow, cyan and purple) visualize the respective DNA origami structures. The Nup96 protein of the nuclear pore complex is depicted in green. The overview image clearly shows the two cellular nuclei. (**c**) Zoom-in of the marked region in **b**. Scale bars, 10 µm (**a**, **b**), 500 nm (**c**).

Supplementary Table 1 | Experimental conditions training set *Digit 1*

Microscope setting	Condition
Microscope	Setup 1
Objective	Apo SR HP TIRF 100x
Camera	Zyla 4.2 Plus
Field of view	512×512 pixel after binning
Frames	15 000
Exposure time	200 ms
Binning	2×2
Tube lens	1×
Excitation laser	561 nm [max power 200 mW]
Laser Power	80 mW

Sample settings	Condition
Sample target	Digit 1 DNA origami
Imager sequence	P1
Imager concentration	1 nM
Imaging buffer	B with PCA/PCD/TX
Dye	Cy3B

Supplementary Table 2 | Experimental conditions in training set *Digit 2*

Setting	Condition
Microscope	Setup 1
Objective	Apo SR HP TIRF 100x
Camera	Zyla 4.2 Plus
Field of view	512×512 pixel after binning
Frames	15 000
Exposure time	200 ms
Binning	2×2
Tube lens	1×
Excitation laser	561 nm [max power 200 mW]
Laser Power	80 mW

Sample settings	Condition
Sample target	Digit 2 DNA origami
Imager sequence	P3
Imager concentration	1 nM
Imaging buffer	B with PCA/PCD/TX
Dye	Cy3B

Supplementary Table 3 | Experimental conditions training set *Digit 3*

Setting	Condition
Microscope	Setup 3
Objective	Apo SR HP TIRF 100x
Camera	Zyla 4.2 Plus
Field of view	512×512 pixel after binning
Frames	15 000
Exposure time	200 ms
Binning	2×2
Tube lens	1×
Excitation laser	560 nm [max power 500 mW]
Laser Power	100 mW

Sample settings	Condition
Sample target	Digit 3 DNA origami
Imager sequence	P5
Imager concentration	1 nM
Imaging buffer	B with PCA/PCD/TX
Dye	Cy3B

Supplementary Table 4 | Experimental conditions in training set *20-nm-grid*

Setting	Condition
Microscope	Setup 3
Objective	Apo SR HP TIRF 100x
Camera	Zyla 4.2 Plus
Field of view	512×512 pixel after binning
Frames	15 000
Exposure time	200 ms
Binning	2×2
Tube lens	1×
Excitation laser	560 nm [max power 500 mW]
Laser Power	100 mW

Sample settings	Condition
Sample target	20-nm-grid DNA origami
Imager sequence	P1
Imager concentration	3 nM
Imaging buffer	B with PCA/PCD/TX
Dye	Cy3B

Supplementary Table 5 | Experimental conditions in validation set

Setting	Condition
Microscope	Setup 3
Objective	Apo SR HP TIRF 100x
Camera	Zyla 4.2 Plus
Field of view	512×512 pixel after binning
Frames	25 000
Exposure time	200 ms
Binning	2×2
Tube lens	1×
Excitation laser	560 nm [max power 500 mW]
Laser Power	100 mW

Sample settings	Condition
Sample target	Digit 1, Digit 2, Digit 3 and 20-nm-grid
Imager sequence	P1, P3, P5
Imager concentration	0,5 nM each
Imaging buffer	B with PCA/PCD/TX
Dye	Cy3B

Supplementary Table 6 | M13mp18 p7249 sequence

TTCCCTTCCTTTCTCGCCACGTTCGCCGGCTTTCCCCGTCAAGCTCTAAATCGGGGGCTCCCTTTAGGGTTCCGATTTAGTGCTTTACGGCACCTCGACCCCAAAAAACT
TGATTTGGGTGATGGTTCACGTAGTGGGCCATCGCCCTGATAGACGGTTTTTCGCCCTTTGACGTTGGAGTCCACGTTCTTTAATAGTGGACTCTTGTTCCAAACTGGAA
CAACACTCAACCCTATCTCGGGCTATTCTTTTGATTTATAAGGGATTTTGCCGATTTCGGAACCACCATCAAACAGGATTTTCGCCTGCTGGGGCAAACCAGCGTGGACC
GCTTGCTGCAACTCTCTCAGGGCCAGGCGGTGAAGGGCAATCAGCTGTTGCCCGTCTCACTGGTGAAAAGAAAAACCACCCTGGCGCCCAATACGCAAACCGCCTCTCCC
CGCGCGTTGGCCGATTCATTAATGCAGCTGGCACGACAGGTTTCCCGACTGGAAAGCGGGCAGTGAGCGCAACGCAATTAATGTGAGTTAGCTCACTCATTAGGCACCCC
AGGCTTTACACTTTATGCTTCCGGCTCGTATGTTGTGTGGAATTGTGAGCGGATAACAATTTCACACAGGAAACAGCTATGACCATGATTACGAATTCGAGCTCGGTACC
CGGGGATCCTCTAGAGTCGACCTGCAGGCATGCAAGCTTGGCACTGGCCGTCGTTTTACAACGTCGTGACTGGGAAAACCCTGGCGTTACCCAACTTAATCGCCTTGCAG
CACATCCCCCTTTCGCCAGCTGGCGTAATAGCGAAGAGGCCCGCACCGATCGCCCTTCCCAACAGTTGCGCAGCCTGAATGGCGAATGGCGCTTTGCCTGGTTTCCGGCA
CCAGAAGCGGTGCCGGAAAGCTGGCTGGAGTGCGATCTTCCTGAGGCCGATACTGTCGTCGTCCCCTCAAACTGGCAGATGCACGGTTACGATGCGCCCATCTACACCAA
CGTGACCTATCCCATTACGGTCAATCCGCCGTTTGTTCCCACGGAGAATCCGACGGGTTGTTACTCGCTCACATTTAATGTTGATGAAAGCTGGCTACAGGAAGGCCAGA
CGCGAATTATTTTTGATGGCGTTCCTATTGGTTAAAAAATGAGCTGATTTAACAAAAATTTAATGCGAATTTTAACAAAATATTAACGTTTACAATTTAAATATTTGCTT
ATACAATCTTCCTGTTTTTGGGGCTTTTCTGATTATCAACCGGGGTACATATGATTGACATGCTAGTTTTACGATTACCGTTCATCGATTCTCTTGTTTGCTCCAGACTC
TCAGGCAATGACCTGATAGCCTTTGTAGATCTCTCAAAAATAGCTACCCTCTCCGGCATTAATTTATCAGCTAGAACGGTTGAATATCATATTGATGGTGATTTGACTGT
CTCCGGCCTTTCTCACCCTTTTGAATCTTTACCTACACATTACTCAGGCATTGCATTTAAAATATATGAGGGTTCTAAAAATTTTTATCCTTGCGTTGAAATAAAGGCTT
CTCCCGCAAAAGTATTACAGGGTCATAATGTTTTTGGTACAACCGATTTAGCTTTATGCTCTGAGGCTTTATTGCTTAATTTTGCTAATTCTTTGCCTTGCCTGTATGAT
TTATTGGATGTTAATGCTACTACTATTAGTAGAATTGATGCCACCTTTTCAGCTCGCGCCCCAAATGAAAATATAGCTAAACAGGTTATTGACCATTTGCGAAATGTATC
TAATGGTCAAACTAAATCTACTCGTTCGCAGAATTGGGAATCAACTGTTATATGGAATGAAACTTCCAGACACCGTACTTTAGTTGCATATTTAAAACATGTTGAGCTAC
AGCATTATATTCAGCAATTAAGCTCTAAGCCATCCGCAAAAATGACCTCTTATCAAAAGGAGCAATTAAAGGTACTCTCTAATCCTGACCTGTTGGAGTTTGCTTCCGGT
CTGGTTCGCTTTGAAGCTCGAATTAAAACGCGATATTTGAAGTCTTTCGGGCTTCCTCTTAATCTTTTTGATGCAATCCGCTTTGCTTCTGACTATAATAGTCAGGGTAA
AGACCTGATTTTTGATTTATGGTCATTCTCGTTTTCTGAACTGTTTAAAGCATTTGAGGGGGATTCAATGAATATTTATGACGATTCCGCAGTATTGGACGCTATCCAGT
CTAAACATTTTACTATTACCCCCTCTGGCAAAACTTCTTTTGCAAAAGCCTCTCGCTATTTTGGTTTTTATCGTCGTCTGGTAAACGAGGGTTATGATAGTGTTGCTCTT
ACTATGCCTCGTAATTCCTTTTGGCGTTATGTATCTGCATTAGTTGAATGTGGTATTCCTAAATCTCAACTGATGAATCTTTCTACCTGTAATAATGTTGTTCCGTTAGT
TCGTTTTATTAACGTAGATTTTTCTTCCCAACGTCCTGACTGGTATAATGAGCCAGTTCTTAAAATCGCATAAGGTAATTCACAATGATTAAAGTTGAAATTAAACCATC
TCAAGCCCAATTTACTACTCGTTCTGGTGTTTCTCGTCAGGGCAAGCCTTATTCACTGAATGAGCAGCTTTGTTACGTTGATTTGGGTAATGAATATCCGGTTCTTGTCA
AGATTACTCTTGATGAAGGTCAGCCAGCCTATGCGCCTGGTCTGTACACCGTTCATCTGTCCTCTTTCAAAGTTGGTCAGTTCGGTTCCCTTATGATTGACCGTCTGCGC
CTCGTTCCGGCTAAGTAACATGGAGCAGGTCGCGGATTTCGACACAATTTATCAGGCGATGATACAAATCTCCGTTGTACTTTGTTTCGCGCTTGGTATAATCGCTGGGG
GTCAAAGATGAGTGTTTTAGTGTATTCTTTTGCCTCTTTCGTTTTAGGTTGGTGCCTTCGTAGTGGCATTACGTATTTTACCCGTTTAATGGAAACTTCCTCATGAAAAA
GTCTTTAGTCCTCAAAGCCTCTGTAGCCGTTGCTACCCTCGTTCCGATGCTGTCTTTCGCTGCTGAGGGTGACGATCCCGCAAAAGCGGCCTTTAACTCCCTGCAAGCCT
CAGCGACCGAATATATCGGTTATGCGTGGGCGATGGTTGTTGTCATTGTCGGCGCAACTATCGGTATCAAGCTGTTTAAGAAATTCACCTCGAAAGCAAGCTGATAAACC
GATACAATTAAAGGCTCCTTTTGGAGCCTTTTTTTTGGAGATTTTCAACGTGAAAAAATTATTATTCGCAATTCCTTTAGTTGTTCCTTTCTATTCTCACTCCGCTGAAA
CTGTTGAAAGTTGTTTAGCAAAATCCCATACAGAAAATTCATTTACTAACGTCTGGAAAGACGACAAAACTTTAGATCGTTACGCTAACTATGAGGGCTGTCTGTGGAAT
GCTACAGGCGTTGTAGTTTGTACTGGTGACGAAACTCAGTGTTACGGTACATGGGTTCCTATTGGGCTTGCTATCCCTGAAAATGAGGGTGGTGGCTCTGAGGGTGGCGG
TTCTGAGGGTGGCGGTTCTGAGGGTGGCGGTACTAAACCTCCTGAGTACGGTGATACACCTATTCCGGGCTATACTTATATCAACCCTCTCGACGGCACTTATCCGCCTG
GTACTGAGCAAAACCCCGCTAATCCTAATCCTTCTCTTGAGGAGTCTCAGCCTCTTAATACTTTCATGTTTCAGAATAATAGGTTCCGAAATAGGCAGGGGGCATTAACT
GTTTATACGGGCACTGTTACTCAAGGCACTGACCCCGTTAAAACTTATTACCAGTACACTCCTGTATCATCAAAAGCCATGTATGACGCTTACTGGAACGGTAAATTCAG
AGACTGCGCTTTCCATTCTGGCTTTAATGAGGATTTATTTGTTTGTGAATATCAAGGCCAATCGTCTGACCTGCCTCAACCTCCTGTCAATGCTGGCGGCGGCTCTGGTG
GTGGTTCTGGTGGCGGCTCTGAGGGTGGTGGCTCTGAGGGTGGCGGTTCTGAGGGTGGCGGCTCTGAGGGAGGCGGTTCCGGTGGTGGCTCTGGTTCCGGTGATTTTGAT
TATGAAAAGATGGCAAACGCTAATAAGGGGGCTATGACCGAAAATGCCGATGAAAACGCGCTACAGTCTGACGCTAAAGGCAAACTTGATTCTGTCGCTACTGATTACGG
TGCTGCTATCGATGGTTTCATTGGTGACGTTTCCGGCCTTGCTAATGGTAATGGTGCTACTGGTGATTTTGCTGGCTCTAATTCCCAAATGGCTCAAGTCGGTGACGGTG
ATAATTCACCTTTAATGAATAATTTCCGTCAATATTTACCTTCCCTCCCTCAATCGGTTGAATGTCGCCCTTTTGTCTTTGGCGCTGGTAAACCATATGAATTTTCTATT
GATTGTGACAAAATAAACTTATTCCGTGGTGTCTTTGCGTTTCTTTTATATGTTGCCACCTTTATGTATGTATTTTCTACGTTTGCTAACATACTGCGTAATAAGGAGTC
TTAATCATGCCAGTTCTTTTGGGTATTCCGTTATTATTGCGTTTCCTCGGTTTCCTTCTGGTAACTTTGTTCGGCTATCTGCTTACTTTTCTTAAAAAGGGCTTCGGTAA
GATAGCTATTGCTATTTCATTGTTTCTTGCTCTTATTATTGGGCTTAACTCAATTCTTGTGGGTTATCTCTCTGATATTAGCGCTCAATTACCCTCTGACTTTGTTCAGG
GTGTTCAGTTAATTCTCCCGTCTAATGCGCTTCCCTGTTTTTATGTTATTCTCTCTGTAAAGGCTGCTATTTTCATTTTTGACGTTAAACAAAAAATCGTTTCTTATTTG
GATTGGGATAAATAATATGGCTGTTTATTTTGTAACTGGCAAATTAGGCTCTGGAAAGACGCTCGTTAGCGTTGGTAAGATTCAGGATAAAATTGTAGCTGGGTGCAAAA
TAGCAACTAATCTTGATTTAAGGCTTCAAAACCTCCCGCAAGTCGGGAGGTTCGCTAAAACGCCTCGCGTTCTTAGAATACCGGATAAGCCTTCTATATCTGATTTGCTT
GCTATTGGGCGCGGTAATGATTCCTACGATGAAAATAAAAACGGCTTGCTTGTTCTCGATGAGTGCGGTACTTGGTTTAATACCCGTTCTTGGAATGATAAGGAAAGACA
GCCGATTATTGATTGGTTTCTACATGCTCGTAAATTAGGATGGGATATTATTTTTCTTGTTCAGGACTTATCTATTGTTGATAAACAGGCGCGTTCTGCATTAGCTGAAC

ATGTTGTTTATTGTCGTCGTCTGGACAGAATTACTTTACCTTTTGTCGGTACTTTATATTCTCTTATTACTGGCTCGAAAATGCCTCTGCCTAAATTACATGTTGGCGTT
GTTAAATATGGCGATTCTCAATTAAGCCCTACTGTTGAGCGTTGGCTTTATACTGGTAAGAATTTGTATAACGCATATGATACTAAACAGGCTTTTTCTAGTAATTATGA
TTCCGGTGTTTATTCTTATTTAACGCCTTATTTATCACACGGTCGGTATTTCAAACCATTAAATTTAGGTCAGAAGATGAAATTAACTAAAATATATTTGAAAAAGTTTT
CTCGCGTTCTTTGTCTTGCGATTGGATTTGCATCAGCATTTACATATAGTTATATAACCCAACCTAAGCCGGAGGTTAAAAAGGTAGTCTCTCAGACCTATGATTTTGAT
AAATTCACTATTGACTCTTCTCAGCGTCTTAATCTAAGCTATCGCTATGTTTTCAAGGATTCTAAGGGAAAATTAATTAATAGCGACGATTTACAGAAGCAAGGTTATTC
ACTCACATATATTGATTTATGTACTGTTTCCATTAAAAAAGGTAATTCAAATGAAATTGTTAAATGTAATTAATTTTGTTTTCTTGATGTTTGTTTCATCATCTTCTTTT
GCTCAGGTAATTGAAATGAATAATTCGCCTCTGCGCGATTTTGTAACTTGGTATTCAAAGCAATCAGGCGAATCCGTTATTGTTTCTCCCGATGTAAAAGGTACTGTTAC
TGTATATTCATCTGACGTTAAACCTGAAAATCTACGCAATTTCTTTATTTCTGTTTTACGTGCAAATAATTTTGATATGGTAGGTTCTAACCCTTCCATTATTCAGAAGT
ATAATCCAAACAATCAGGATTATATTGATGAATTGCCATCATCTGATAATCAGGAATATGATGATAATTCCGCTCCTTCTGGTGGTTTCTTTGTTCCGCAAAATGATAAT
GTTACTCAAACTTTTAAAATTAATAACGTTCGGGCAAAGGATTTAATACGAGTTGTCGAATTGTTTGTAAAGTCTAATACTTCTAAATCCTCAAATGTATTATCTATTGA
CGGCTCTAATCTATTAGTTGTTAGTGCTCCTAAAGATATTTTAGATAACCTTCCTCAATTCCTTTCAACTGTTGATTTGCCAACTGACCAGATATTGATTGAGGGTTTGA
TATTTGAGGTTCAGCAAGGTGATGCTTTAGATTTTTCATTTGCTGCTGGCTCTCAGCGTGGCACTGTTGCAGGCGGTGTTAATACTGACCGCCTCACCTCTGTTTTATCT
TCTGCTGGTGGTTCGTTCGGTATTTTTAATGGCGATGTTTTAGGGCTATCAGTTCGCGCATTAAAGACTAATAGCCATTCAAAAATATTGTCTGTGCCACGTATTCTTAC
GCTTTCAGGTCAGAAGGGTTCTATCTCTGTTGGCCAGAATGTCCCTTTTATTACTGGTCGTGTGACTGGTGAATCTGCCAATGTAAATAATCCATTTCAGACGATTGAGC
GTCAAAATGTAGGTATTTCCATGAGCGTTTTTCCTGTTGCAATGGCTGGCGGTAATATTGTTCTGGATATTACCAGCAAGGCCGATAGTTTG

Supplementary Table 7 | Rectangular DNA origami staple strands

Plate	Pos	Name	Sequence	Digit 1	Digit 2	Digit 3	20-nm-grid
1	A1	21[32]23[31]BLK	TTTTCACTCAAAGGGCGAAAAACCATCACC				
1	A2	19[32]21[31]BLK	GTCGACTTCGGCCAACGCGCGGGGTTTTTC				
1	A3	17[32]19[31]BLK	TGCATCTTTCCCAGTCACGACGGCCTGCAG				
1	A4	15[32]17[31]BLK	TAATCAGCGGATTGACCGTAATCGTAACCG				
1	A5	13[32]15[31]BLK	AACGCAAAATCGATGAACGGTACCGGTTGA				
1	A6	11[32]13[31]BLK	AACAGTTTTGTACCAAAAACATTTTATTTC				
1	A7	9[32]11[31]BLK	TTTACCCCAACATGTTTTAAATTTCCATAT				
1	A8	7[32]9[31]BLK	TTTAGGACAAATGCTTTAAACAATCAGGTC				
1	A9	5[32]7[31]BLK	CATCAAGTAAAACGAACTAACGAGTTGAGA				
1	A10	3[32]5[31]BLK	AATACGTTTGAAAGAGGACAGACTGACCTT				
1	A11	1[32]3[31]BLK	AGGCTCCAGAGGCTTTGAGGACACGGGTAA				
1	A12	0[47]1[31]BLK	AGAAAGGAACAACTAAAGGAATTCAAAAAAA				
1	B1	23[32]22[48]BLK	CAAATCAAGTTTTTTGGGGTCGAAACGTGGA				
1	B2	22[47]20[48]BLK	CTCCAACGCAGTGAGACGGGCAACCAGCTGCA				
1	B3	20[47]18[48]BLK	TTAATGAACTAGAGGATCCCCGGGGGGTAACG				P1
1	B4	18[47]16[48]BLK	CCAGGGTTGCCAGTTTGAGGGGACCCGTGGGA				
1	B5	16[47]14[48]BLK	ACAAACGGAAAAGCCCCAAAAACACTGGAGCA				
1	B6	14[47]12[48]BLK	AACAAGAGGGATAAAAATTTTTAGCATAAAGC				
1	B7	12[47]10[48]BLK	TAAATCGGGATTCCCAATTCTGCGATATAATG				P1
1	B8	10[47]8[48]BLK	CTGTAGCTTGACTATTATAGTCAGTTCATTGA				
1	B9	8[47]6[48]BLK	ATCCCCCTATACCACATTCAACTAGAAAAATC				
1	B10	6[47]4[48]BLK	TACGTTAAAGTAATCTTGACAAGAACCGAACT				
1	B11	4[47]2[48]BLK	GACCAACTAATGCCACTACGAAGGGGGTAGCA				P1
1	B12	2[47]0[48]BLK	ACGGCTACAAAAGGAGCCTTTAATGTGAGAAT				
1	C1	21[56]23[63]BLK	AGCTGATTGCCCTTCAGAGTCCACTATTAAAGGGTGCCGT				
1	C4	15[64]18[64]BLK	GTATAAGCCAACCCGTCGGATTCTGACGACAGTATCGGCCGCAAGGCG				
1	C5	13[64]15[63]BLK	TATATTTTGTCATTGCCTGAGAGTGGAAGATT				
1	C6	11[64]13[63]BLK	GATTTAGTCAATAAAGCCTCAGAGAACCCTCA				

1	C7	9[64]11[63]BLK	CGGATTGCAGAGCTTAATTGCTGAAACGAGTA				
1	C8	7[56]9[63]BLK	ATGCAGATACATAACGGGAATCGTCATAAATAAAGCAAAG				
1	C11	1[64]4[64]BLK	TTTATCAGGACAGCATCGGAACGACACCAACCTAAAACGAGGTCAATC				
1	C12	0[79]1[63]BLK	ACAACTTTCAACAGTTTCAGCGGATGTATCGG				
1	D1	23[64]22[80]BLK	AAAGCACTAAATCGGAACCCTAATCCAGTT			P5	
1	D2	22[79]20[80]BLK	TGGAACAACCGCCTGGCCCTGAGGCCCGCT			P5	
1	D3	20[79]18[80]BLK	TTCCAGTCGTAATCATGGTCATAAAAGGGG			P5	
1	D4	18[79]16[80]BLK	GATGTGCTTCAGGAAGATCGCACAATGTGA		P3	P5	
1	D5	16[79]14[80]BLK	GCGAGTAAAAATATTTAAATTGTTACAAAG		P3	P5	
1	D6	14[79]12[80]BLK	GCTATCAGAAATGCAATGCCTGAATTAGCA	P1	P3		
1	D7	12[79]10[80]BLK	AAATTAAGTTGACCATTAGATACTTTTGCG	P1	P3		
1	D8	10[79]8[80]BLK	GATGGCTTATCAAAAAGATTAAGAGCGTCC				
1	D9	8[79]6[80]BLK	AATACTGCCCAAAAGGAATTACGTGGCTCA				
1	D10	6[79]4[80]BLK	TTATACCACCAAATCAACGTAACGAACGAG				
1	D11	4[79]2[80]BLK	GCGCAGACAAGAGGCAAAAGAATCCCTCAG				
1	D12	2[79]0[80]BLK	CAGCGAAACTTGCTTTCGAGGTGTTGCTAA				
1	E1	21[96]23[95]BLK	AGCAAGCGTAGGGTTGAGTGTTGTAGGGAGCC				
1	E2	19[96]21[95]BLK	CTGTGTGATTGCGTTGCGCTCACTAGAGTTGC				
1	E3	17[96]19[95]BLK	GCTTTCCGATTACGCCAGCTGGCGGCTGTTTC				
1	E4	15[96]17[95]BLK	ATATTTTGGCTTTCATCAACATTATCCAGCCA		P3		
1	E5	13[96]15[95]BLK	TAGGTAAACTATTTTTGAGAGATCAAACGTTA				
1	E6	11[96]13[95]BLK	AATGGTCAACAGGCAAGGCAAAGAGTAATGTG	P1			
1	E7	9[96]11[95]BLK	CGAAAGACTTTGATAAGAGGTCATATTTCGCA			P5	
1	E8	7[96]9[95]BLK	TAAGAGCAAATGTTTAGACTGGATAGGAAGCC	P1	P3		
1	E9	5[96]7[95]BLK	TCATTCAGATGCGATTTTAAGAACAGGCATAG				
1	E10	3[96]5[95]BLK	ACACTCATCCATGTTACTTAGCCGAAAGCTGC				
1	E11	1[96]3[95]BLK	AAACAGCTTTTTGCGGGATCGTCAACACTAAA				
1	E12	0[111]1[95]BLK	TAAATGAATTTTCTGTATGGGATTAATTTCTT				
1	F1	23[96]22[112]BLK	CCCGATTTAGAGCTTGACGGGGAAAAAGAATA				
1	F2	22[111]20[112]BLK	GCCCGAGAGTCCACGCTGGTTTGCAGCTAACT				
1	F3	20[111]18[112]BLK	CACATTAAAATTGTTATCCGCTCATGCGGGCC		P3		P1
1	F4	18[111]16[112]BLK	TCTTCGCTGCACCGCTTCTGGTGCGGCCTTCC				
1	F5	16[111]14[112]BLK	TGTAGCCATTAAAATTCGCATTAAATGCCGGA	P1			
1	F6	14[111]12[112]BLK	GAGGGTAGGATTCAAAAGGGTGAGACATCCAA				
1	F7	12[111]10[112]BLK	TAAATCATATAACCTGTTTAGCTAACCTTTAA	P1		P5	P1
1	F8	10[111]8[112]BLK	TTGCTCCTTTCAAATATCGCGTTTGAGGGGGT		P3		
1	F9	8[111]6[112]BLK	AATAGTAAACACTATCATAACCCTCATTGTGA				
1	F10	6[111]4[112]BLK	ATTACCTTTGAATAAGGCTTGCCCAAATCCGC				
1	F11	4[111]2[112]BLK	GACCTGCTCTTTGACCCCCAGCGAGGGAGTTA				P1
1	F12	2[111]0[112]BLK	AAGGCCGCTGATACCGATAGTTGCGACGTTAG				
1	G1	21[120]23[127]BLK	CCCAGCAGGCGAAAAATCCCTTATAAATCAAGCCGGCG				
1	G4	15[128]18[128]BLK	TAAATCAAAATAATTCGCGTCTCGGAAACCAGGCAAAGGGAAGG				
1	G5	13[128]15[127]BLK	GAGACAGCTAGCTGATAAATTAATTTTTGT	P1			
1	G6	11[128]13[127]BLK	TTTGGGGATAGTAGTAGCATTAAAAGGCCG				
1	G7	9[128]11[127]BLK	GCTTCAATCAGGATTAGAGAGTTATTTTCA			P5	
1	G8	7[120]9[127]BLK	CGTTTACCAGACGACAAAGAAGTTTTGCCATAATTCGA	P1	P3		
1	G11	1[128]4[128]BLK	TGACAACTCGCTGAGGCTTGCATTATACCAAGCGCGATGATAAA				
1	G12	0[143]1[127]BLK	TCTAAAGTTTTGTCGTCTTTCCAGCCGACAA				

1	H1	21[160]22[144]BLK	TCAATATCGAACCTCAAATATCAATTCCGAAA				
1	H2	19[160]20[144]BLK	GCAATTCACATATTCCTGATTATCAAAGTGTA				
1	H3	17[160]18[144]BLK	AGAAAACAAAGAAGATGATGAAACAGGCTGCG				
1	H4	15[160]16[144]BLK	ATCGCAAGTATGTAAATGCTGATGATAGGAAC	P1			
1	H5	13[160]14[144]BLK	GTAATAAGTTAGGCAGAGGCATTTATGATATT				
1	H6	11[160]12[144]BLK	CCAATAGCTCATCGTAGGAATCATGGCATCAA			P5	
1	H7	9[160]10[144]BLK	AGAGAGAAAAAAATGAAAATAGCAAGCAAACT	P1	P3		
1	H8	7[160]8[144]BLK	TTATTACGAAGAACTGGCATGATTGCGAGAGG				
1	H9	5[160]6[144]BLK	GCAAGGCCTCACCAGTAGCACCATGGGCTTGA				
1	H10	3[160]4[144]BLK	TTGACAGGCCACCACCAGAGCCGCGATTTGTA				
1	H11	1[160]2[144]BLK	TTAGGATTGGCTGAGACTCCTCAATAACCGAT				
1	H12	0[175]0[144]BLK	TCCACAGACAGCCCTCATAGTTAGCGTAACGA				
2	A1	23[128]23[159]BLK	AACGTGGCGAGAAAGGAAGGGAAACCAGTAA			P5	
2	A2	22[143]21[159]BLK	TCGGCAAATCCTGTTTGATGGTGGACCCTCAA			P5	
2	A3	20[143]19[159]BLK	AAGCCTGGTACGAGCCGGAAGCATAGATGATG			P5	
2	A4	18[143]17[159]BLK	CAACTGTTGCGCCATTCGCCATTCAAACATCA	P1		P5	
2	A5	16[143]15[159]BLK	GCCATCAAGCTCATTTTTTAACCACAAATCCA			P5	
2	A6	14[143]13[159]BLK	CAACCGTTTCAAATCACCATCAATTCGAGCCA			P5	
2	A7	12[143]11[159]BLK	TTCTACTACGCGAGCTGAAAAGGTTACCGCGC		P3		
2	A8	10[143]9[159]BLK	CCAACAGGAGCGAACCAGACCGGAGCCTTTAC	P1			
2	A9	8[143]7[159]BLK	CTTTTGCAGATAAAAACCAAAATAAAGACTCC				
2	A10	6[143]5[159]BLK	GATGGTTTGAACGAGTAGTAAATTTACCATTA				
2	A11	4[143]3[159]BLK	TCATCGCCAACAAAGTACAACGGACGCCAGCA				
2	A12	2[143]1[159]BLK	ATATTCGGAACCATCGCCCACGCAGAGAAGGA				
2	B1	23[160]22[176]BLK	TAAAAGGGACATTCTGGCCAACAAAGCATC				
2	B2	22[175]20[176]BLK	ACCTTGCTTGGTCAGTTGGCAAAGAGCGGA				
2	B3	20[175]18[176]BLK	ATTATCATTCAATATAATCCTGACAATTAC				P1
2	B4	18[175]16[176]BLK	CTGAGCAAAAATTAATTACATTTTGGGTTA				
2	B5	16[175]14[176]BLK	TATAACTAACAAAGAACGCGAGAACGCCAA		P3		
2	B6	14[175]12[176]BLK	CATGTAATAGAATATAAAGTACCAAGCCGT		P3	P5	
2	B7	12[175]10[176]BLK	TTTTATTTAAGCAAATCAGATATTTTTTGT	P1			P1
2	B8	10[175]8[176]BLK	TTAACGTCTAACATAAAAACAGGTAACGGA				
2	B9	8[175]6[176]BLK	ATACCCAACAGTATGTTAGCAAATTAGAGC				
2	B10	6[175]4[176]BLK	CAGCAAAAGGAAACGTCACCAATGAGCCGC				
2	B11	4[175]2[176]BLK	CACCAGAAAGGTTGAGGCAGGTCATGAAAG				P1
2	B12	2[175]0[176]BLK	TATTAAGAAGCGGGGTTTTGCTCGTAGCAT				
2	C1	21[184]23[191]BLK	TCAACAGTTGAAAGGAGCAAATGAAAAATCTAGAGATAGA				
2	C4	15[192]18[192]BLK	TCAAATATAACCTCCGGCTTAGGTAACAATTTCATTTGAAGGCGAATT				
2	C5	13[192]15[191]BLK	GTAAAGTAATCGCCATATTTAACAAAACTTTT		P3		
2	C6	11[192]13[191]BLK	TATCCGGTCTCATCGAGAACAAGCGACAAAAG				
2	C7	9[192]11[191]BLK	TTAGACGGCCAAATAAGAAACGATAGAAGGCT			P5	
2	C8	7[184]9[191]BLK	CGTAGAAAATACATACCGAGGAAACGCAATAAGAAGCGCA	P1			
2	C11	1[192]4[192]BLK	GCGGATAACCTATTATTCTGAAACAGACGATTGGCCTTGAAGAGCCAC				
2	C12	0[207]1[191]BLK	TCACCAGTACAAACTACAACGCCTAGTACCAG				
2	D1	23[192]22[208]BLK	ACCCTTCTGACCTGAAAGCGTAAGACGCTGAG				
2	D2	22[207]20[208]BLK	AGCCAGCAATTGAGGAAGGTTATCATCATTTT				
2	D3	20[207]18[208]BLK	GCGGAACATCTGAATAATGGAAGGTACAAAAT		P3		
2	D4	18[207]16[208]BLK	CGCGCAGATTACCTTTTTTAATGGGAGAGACT		P3		

2	D5	16[207]14[208]BLK	ACCTTTTTATTTTAGTTAATTTCATAGGGCTT				
2	D6	14[207]12[208]BLK	AATTGAGAATTCTGTCCAGACGACTAAACCAA				
2	D7	12[207]10[208]BLK	GTACCGCAATTCTAAGAACGCGAGTATTATTT	P1		P5	
2	D8	10[207]8[208]BLK	ATCCCAATGAGAATTAACTGAACAGTTACCAG				
2	D9	8[207]6[208]BLK	AAGGAAACATAAAGGTGGCAACATTATCACCG				
2	D10	6[207]4[208]BLK	TCACCGACGCACCGTAATCAGTAGCAGAACCG				
2	D11	4[207]2[208]BLK	CCACCCTCTATTCACAAACAAATACCTGCCTA				
2	D12	2[207]0[208]BLK	TTTCGGAAGTGCCGTCGAGAGGGTGAGTTTCG				
2	E1	21[224]23[223]BLK	CTTTAGGGCCTGCAACAGTGCCAATACGTG				
2	E2	19[224]21[223]BLK	CTACCATAGTTTGAGTAACATTTAAAATAT				
2	E3	17[224]19[223]BLK	CATAAATCTTTGAATACCAAGTGTTAGAAC		P3		
2	E4	15[224]17[223]BLK	CCTAAATCAAAATCATAGGTCTAAACAGTA		P3		
2	E5	13[224]15[223]BLK	ACAACATGCCAACGCTCAACAGTCTTCTGA		P3		
2	E6	11[224]13[223]BLK	GCGAACCTCCAAGAACGGGTATGACAATAA		P3		
2	E7	9[224]11[223]BLK	AAAGTCACAAAATAAACAGCCAGCGTTTTA		P3	P5	
2	E8	7[224]9[223]BLK	AACGCAAAGATAGCCGAACAAACCCTGAAC	P1	P3		
2	E9	5[224]7[223]BLK	TCAAGTTTCATTAAAGGTGAATATAAAAGA		P3		
2	E10	3[224]5[223]BLK	TTAAAGCCAGAGCCGCCACCCTCGACAGAA				
2	E11	1[224]3[223]BLK	GTATAGCAAACAGTTAATGCCCAATCCTCA				
2	E12	0[239]1[223]BLK	AGGAACCCATGTACCGTAACACTTGATATAA				
2	F1	23[224]22[240]BLK	GCACAGACAATATTTTTGAATGGGGTCAGTA			P5	
2	F2	22[239]20[240]BLK	TTAACACCAGCACTAACAACTAATCGTTATTA			P5	
2	F3	20[239]18[240]BLK	ATTTTAAAATCAAAATTATTTGCACGGATTCG			P5	P1
2	F4	18[239]16[240]BLK	CCTGATTGCAATATATGTGAGTGATCAATAGT			P5	
2	F5	16[239]14[240]BLK	GAATTTATTTAATGGTTTGAAATATTCTTACC			P5	
2	F6	14[239]12[240]BLK	AGTATAAAGTTCAGCTAATGCAGATGTCTTTC			P5	
2	F7	12[239]10[240]BLK	CTTATCATTCCCGACTTGCGGGAGCCTAATTT	P1			P1
2	F8	10[239]8[240]BLK	GCCAGTTAGAGGGTAATTGAGCGCTTTAAGAA				
2	F9	8[239]6[240]BLK	AAGTAAGCAGACACCACGGAATAATATTGACG				
2	F10	6[239]4[240]BLK	GAAATTATTGCCTTTAGCGTCAGACCGGAACC				
2	F11	4[239]2[240]BLK	GCCTCCCTCAGAATGGAAAGCGCAGTAACAGT				P1
2	F12	2[239]0[240]BLK	GCCCGTATCCGGAATAGGTGTATCAGCCCAAT				
2	G1	21[248]23[255]BLK	AGATTAGAGCCGTCAAAAAACAGAGGTGAGGCCTATTAGT				
2	G4	15[256]18[256]BLK	GTGATAAAAAGACGCTGAGAAGAGATAACCTTGCTTCTGTTCGGGAGA				
2	G5	13[256]15[255]BLK	GTTTATCAATATGCGTTATACAAACCGACCGT				
2	G6	11[256]13[255]BLK	GCCTTAAACCAATCAATAATCGGCACGCGCCT				
2	G7	9[256]11[255]BLK	GAGAGATAGAGCGTCTTTCCAGAGGTTTTGAA				
2	G8	7[248]9[255]BLK	GTTTATTTTGTCACAATCTTACCGAAGCCCTTTAATATCA	P1			
2	G11	1[256]4[256]BLK	CAGGAGGTGGGGTCAGTGCCTTGAGTCTCTGAATTTACCGGGAACCAG				
2	G12	0[271]1[255]BLK	CCACCCTCATTTTCAGGGATAGCAACCGTACT				
2	H1	23[256]22[272]BLK	CTTTAATGCGCGAACTGATAGCCCCACCAG				
2	H2	22[271]20[272]BLK	CAGAAGATTAGATAATACATTTGTCGACAA				
2	H3	20[271]18[272]BLK	CTCGTATTAGAAATTGCGTAGATACAGTAC				
2	H4	18[271]16[272]BLK	CTTTTACAAAATCGTCGCTATTAGCGATAG				
2	H5	16[271]14[272]BLK	CTTAGATTTAAGGCGTTAAATAAAGCCTGT				
2	H6	14[271]12[272]BLK	TTAGTATCACAATAGATAAGTCCACGAGCA				
2	H7	12[271]10[272]BLK	TGTAGAAATCAAGATTAGTTGCTCTTACCA				
2	H8	10[271]8[272]BLK	ACGCTAACACCCACAAGAATTGAAAATAGC				

2	H9	8[271]6[272]BLK	AATAGCTATCAATAGAAAATTCAACATTCA				
2	H10	6[271]4[272]BLK	ACCGATTGTCGGCATTTTCGGTCATAATCA				
2	H11	4[271]2[272]BLK	AAATCACCTTCCAGTAAGCGTCAGTAATAA				
2	H12	2[271]0[272]BLK	GTTTTAACTTAGTACCGCCACCCAGAGCCA				

Supplementary Table 8 | Biotinylated staple strands

Position	Name	Sequence	Modification
C02	18[63]20[56]BIOTIN	ATTAAGTTTACCGAGCTCGAATTCGGGAAACCTGTCGTGC	5' - Biotin
C09	4[63]6[56]BIOTIN	ATAAGGGAACCGGATATTCATTACGTCAGGACGTTGGGAA	5' - Biotin
G02	18[127]20[120]BIOTIN	GCGATCGGCAATTCCACACAACAGGTGCCTAATGAGTG	5' - Biotin
G09	4[127]6[120]BIOTIN	TTGTGTCGTGACGAGAAACACCAAATTTCAACTTTAAT	5' - Biotin
K02	18[191]20[184]BIOTIN	ATTCATTTTTGTTTGGATTATACTAAGAAACCACCAGAAG	5' - Biotin
K09	4[191]6[184]BIOTIN	CACCCTCAGAAACCATCGATAGCATTGAGCCATTTGGGAA	5' - Biotin
O02	18[255]20[248]BIOTIN	AACAATAACGTAAAACAGAAATAAAAATCCTTTGCCCGAA	5' - Biotin
O09	4[255]6[248]BIOTIN	AGCCACCACTGTAGCGCGTTTTCAAGGGAGGGAAGGTAAA	5' - Biotin

Supplementary Table 9 | DNA-PAINT docking site sequences

Name	Sequence	Modification
P1 docking strand	TTATACATCTA	-
P3 docking strand	TTTCTTCATTA	-
P5 docking strand	TTCAATGTATG	-

Supplementary Table 10 | DNA-PAINT imager sequences

Name	Sequence	Modification
Imager P1	CTAGATGTAT	3' – Cy3B
Imager P3	GTAATGAAGA	3' – Cy3B
Imager P5	CATACATTGA	3' – Cy3B

nanoTRON Train: *Digit 1*, *Digit 2*, *Digit 3* and *20 nm grid* DNA origami

Computer	CPU	Cores	Total runtime	Runtime per epoch
MacBook Pro 13 (early-2015)	Intel® Core™ i5-5257U @ 2.70GHz	2	~ 30 min	~ 1.1 min
MacBook Pro 15 (mid-2014)	Intel® Core™ i7-4980HQ @ 2.80GHz	4	~ 15 min	~ 0.5 min
Dell XPS 15 (9550)	Intel® Core™ i7-6700HQ @ 2.60GHz	4	~ 37 min	~ 1.3 min
Dell Precision T7910	2x Intel® Xeon® E5-2680 v3 @ 2.50GHz	24	~ 47 min	~ 1.7 min
Dell Precision T7910	2x Intel® Xeon® E5-2660 v3 @ 2.60GHz	20	~ 25 min	~ 0.9 min

Supplementary Table 11 | Training runtime comparison with various computers. The runtime for training of the 1-layer MLP with 550 nodes and the training data from **Supplementary Figure 4-7** was recorded on different computer systems. Three mobile devices and two high-performance workstations. Computation time ranges from 15 – 47 minutes. The training was performed using 247522 grayscale images.

nanoTRON Predict: 13332 nanopatterns with *Digit 1*, *Digit 2*, *Digit 3* and *20 nm grid* DNA origami

Computer	CPU	Cores	Runtime
MacBook Pro 13 (early-2015)	Intel® Core™ i5-5257U @ 2.70GHz	2	~ 9.3 min
MacBook Pro 15 (mid-2014)	Intel® Core™ i7-4980HQ @ 2.80GHz	4	~ 5.6 min
Dell XPS 15	Intel® Core™ i7-6700HQ @ 2.60GHz	4	~ 4.9 min
Dell Precision T7910	2x Intel® Xeon® E5-2680 v3 @ 2.50GHz	24	~ 4.2 min
Dell Precision T7910	2x Intel® Xeon® E5-2660 v3 @ 2.60GHz	20	~ 3.4 min

Supplementary Table 12 | Prediction runtime comparison with various computers. The runtime for prediction of the validation data set with four unique DNA origami nanopatterns (**Figure 1c**) was recorded on different computer systems. The nanoTRON model described in **Supplementary Text 2** was used. 13332 nanopatterns were classified between 3.4 – 9.3 minutes.

Training with *Digit 1*, *Digit 2*, *Digit 3* and *20 nm grid* DNA origami

Neural network	Layout	Processed with	Total runtime	Runtime per epoch	Train accuracy	Test accuracy
nanoTRON MLP	1-layer FC 550 nodes	CPU	~ 47 min	~ 1.7 min	~ 0.99	~ 0.98
Keras LeNet-5	7-layer CNN	CPU	~ 36 h	~ 53 min	~ 0.99	~ 0.98
		GPU	~ 12 min	~ 0.3 min	~ 0.99	~ 0.98

CPU: 2x Intel® Xeon® E5-2680 v3 @ 2.50GHz (24 cores)
GPU: NVIDIA GeForce GTX 1080 Ti

Supplementary Table 13 | nanoTRON MLP compared with LeNet-5 CNN. Runtime and performance evaluation of the nanoTRON 1-layer perceptron described in **Supplementary Text 2** and the LeNet-5 convolutional neural network (CNN) (Lecun, et al., 1998) implemented in Keras (Chollet, 2015). The 7-layer CNN network design is listed in **Supplementary Table 14**. For the comparison, the augmented training data from **Supplementary Text 2** was used. The networks were trained in total with 247522 grayscale images. Input shape was 50 x 50 pixels with gray values from 0 to 1. Early stop callback was monitoring validation accuracy (10% split of training data) with a minimum change of 1E-4 over at least 10 epochs. Solver was set in all cases to "adam". Both neural networks classified the test set of 74257 images with a test accuracy of around ~ 0.98. nanoTRON MLP reached the early stop after ~ 47 minutes with CPU processing, while the training of the LeNet-5 CNN lasted almost 1.5 days using the CPU. The same network trained with the high-performance GPU finished after ~ 12 min. This implies that CNN training is practically only feasible using GPU processing.

Layer type	Layer configuration	Output shape	Parameter #
Conv2D	Filter 6, Kernel 5, Stride 1, tanh	(None, 50, 50, 6)	156
Average Pooling 2D	Pool 2, Stride 1	(None, 25, 25, 6)	0
Conv2D	Filter 16, Kernel 5, Stride 1, tanh	(None, 21, 21, 16)	2416
Average Pooling 2D	Pool 2, Stride 1	(None, 10, 10, 16)	0
Conv2D	Filter 120, Kernel 5, Stride 1, tanh	(None, 6, 6, 120)	48120
Flatten		(None, 4320)	0
Dense	Units 84, tanh	(None, 84)	362964
Dense	Units 4, softmax	(None, 4)	340

Total parameters: 413,996
Trainable parameters: 413,996
Non-trainable parameters: 0

Supplementary Table 14 | LeNet-5 CNN Design. Convolutional neural network model design of the LeNet-5 implemented in Keras and used for comparison with the nanoTRON 1-layer MLP, described in **Supplementary Text 2**.

Materials and buffers. Unmodified DNA oligonucleotides, fluorescently modified DNA oligonucleotides and biotinylated DNA oligonucleotides were purchased from MWG Eurofins. M13mp18 scaffold was obtained from Tilibit. BSA-Biotin was obtained from Sigma-Aldrich (cat: A8549). Streptavidin was ordered from Invitrogen (cat: S-888). Tris 1M pH 8.0 (cat: AM9856), EDTA 0.5M pH 8.0 (cat: AM9261), Magnesium 1M (cat: AM9530G) and Sodium Chloride 5M (cat: AM9759) were ordered from Ambion. Ultrapure water (cat: 10977-035) was purchased from Gibco. Polyethylene glycol (PEG)-8000 (catalog no. 6510-1KG) was purchased from Merck. Glass slides (cat: 48811-703) were obtained from VWR. Coverslips were purchased from Marienfeld (cat: 0107032). Silicon (cat.1300 1000) was ordered from picodent. Double sided tape (cat: 665D) was ordered from Scotch.

Two buffers were used for sample preparation and imaging:

- Buffer A (10 mM Tris-HCl pH 7.5, 100 mM NaCl, 0.05% Tween 20, pH 7.5)
- Buffer B (5 mM Tris-HCl pH 8, 10 mM $MgCl_2$, 1 mM EDTA, 0.05% Tween 20, pH 8).
- Imaging Buffer B was supplemented with: 1× Trolox, 1× PCA and 1× PCD (see paragraph below for details). This photo-stabilization system allowed us to maximize the number of photons per event and thus achieve optimal spatial resolution.

Trolox, PCA and PCD stocks:

- 100× Trolox: 100mg Trolox, 430µl 100% methanol, 345 µl 1M NaOH in 3.2ml H2O.
- 40× PCA: 154mg PCA, 10ml water and NaOH were mixed and the pH was adjusted to 9.0.
- 100× PCD: 9.3mg PCD, 13.3ml of buffer was used (100 mM Tris-HCl pH 8, 50 mM KCl, 1mM EDTA, 50% glycerol).

Optical setups.

Super-resolution setup 1: Fluorescence imaging was partly carried out (see Imaging conditions) on an inverted microscope (Nikon Instruments, Eclipse Ti) with the Perfect Focus System, applying an objective-type TIRF configuration with an oil-immersion objective (Nikon Instruments, Apo SR HP TIRF ×100, numerical aperture 1.49, Oil). A 561nm (Coherent Sapphire, 200 mW, DPSS-system) laser was used for excitation. The laser beam was passed through cleanup filters (Chroma Technology, ZET561/10) and coupled into the microscope objective using a beam splitter (Chroma Technology, ZT561rdc). Fluorescence light was spectrally filtered with an emission filter (Chroma Technology, ET600/50m and ET575lp) and imaged on a sCMOS camera (Andor, Zyla 4.2 Plus) without further magnification, resulting in an effective pixel size of 130nm (after 2×2 binning).

Super-resolution setup 3: Fluorescence imaging was partly carried out (see Imaging conditions) on an inverted microscope (Nikon Instruments, Eclipse Ti2) with the Perfect Focus System, applying an objective-type TIRF configuration with an oil-immersion objective (Nikon Instruments, Apo SR HP TIRF ×100, numerical aperture 1.49, Oil). A 560 nm (MPB Communications Inc., 500 mW, DPSS-system) laser was used for excitation. The laser beam was passed through cleanup filters (Chroma Technology, ZET561/10) and coupled into the microscope objective using a beam splitter (Chroma Technology, ZT561rdc). Fluorescence light was spectrally filtered with an emission filter (Chroma Technology, ET600/50m and ET575lp) and imaged on a sCMOS camera (Andor, Zyla 4.2 Plus) without further magnification, resulting in an effective pixel size of 130nm (after 2×2 binning).

DNA origami self-assembly. The Rothemund rectangular origami (RRO) from **Figure 1** were synthesized in a one-pot reaction with 50 µl total volume containing 10 nM scaffold strand (M13mp18), 100 nM core staples, 1 µM biotinylated staples and 1 µM DNA-PAINT handles. Sequences are listed in **Supplementary Table 6-9**. The folding buffer was 1x TE buffer with 12.5 mM $MgCl_2$. Structures were annealed using a thermal ramp. First, incubating for 5 min at 80°C, then going from 65°C to 4°C over the course of 3 hours. DNA origami

structures were purified via two rounds of PEG precipitation by adding the same volume of PEG-buffer, centrifuging at 14,000g at 4°C for 30min, removing the supernatant and resuspending in folding buffer.

Nanobody conjugation. Unconjugated GFP Nanobody (Fluotag-Q anti-GFP) was purchased from Nanotag. The nanobody DNA conjugation was performed according to the protocol described before (Schlichthaerle, et al., 2018).

Super-resolution DNA-PAINT imaging with DNA origami. For chamber preparation, a piece of coverslip (no. 1.5, 18 × 18 mm, ~0.17 mm thick) and a glass slide (76 × 26 mm, 1 mm thick) were sandwiched together by two strips of double-sided tape to form a flow chamber with inner volume of ~20 µl. First, 20 µl of biotin-labeled bovine albumin (1 mg/ml, dissolved in buffer A) was flown into the chamber and incubated for 2 min. Then the chamber was washed using 40 µl of buffer A. Second, 20 µl of streptavidin (0.5mg/ml, dissolved in buffer A) was then flown through the chamber and incubated for 2 min. Next, the chamber was washed with 20 µl of buffer A and subsequently with 20 µl of buffer B. Then ~500 pM of the DNA origami structures (RRO) were flown into the chamber and allowed to attach to the surface for 2 min. Finally, the imaging buffer with buffer B with dye-labeled imager strands was flowed into the chamber and sealed with silicon. Imaging conditions are listed in **Supplementary Table 1-5**. Imager sequences are stated in **Supplementary Table 10**.

Super-resolution DNA-PAINT imaging with nuclear pore complex. Nuclear Pore Complex (NPC) imaging was performed using a U2OS cell line genetically modified with an EGFP fused to Nup96 proteins. The cells were fixed in 2.4% paraformaldehyde in PBS for 30 min. After fixation, cells were washed three times with PBS followed by permeabilization with 0.25% Triton-X-100 in PBS for 5 min. Then, cells were blocked in blocking buffer (3% BSA + 0.02% Tween-20) for 60 min. Anti-GFP nanobody conjugated to a DNA-PAINT docking site was diluted in blocking buffer to approximately 25 nM and incubated overnight at 4°C. On the next day, cells were washed 2x with PBS followed by an incubation with gold nanoparticles for 5 min. Cells were washed two times with PBS, then the imaging solution (PBS + 500 mM NaCl) was added containing 250 pM Cy3B labeled imager strands (Schueder, et al., 2019).

Super-resolution reconstruction. Raw fluorescence data was subjected to spot-finding and subsequent super-resolution reconstruction using the Picasso software package. The drift correction was performed with a redundant cross-correlation (segmentation: 1000) and subsequently *Undrift from picked* with all picked DNA origami structures. The DNA origami were picked using Picasso *Pick Tool* and *Pick similar*.

References

Belthangady, C. and Royer, L.A. Applications, promises, and pitfalls of deep learning for fluorescence image reconstruction. *Nature Methods* 2019;16(12):1215-1225.

Chollet, F.a.o. 2015. Keras

Hahnloser, R.H., *et al.* Digital selection and analogue amplification coexist in a cortex-inspired silicon circuit. *Nature* 2000;405(6789):947-951.

He, K.M., *et al.* Delving Deep into Rectifiers: Surpassing Human-Level Performance on ImageNet Classification. *Ieee I Conf Comp Vis* 2015:1026-1034.

Jungmann, R., *et al.* Single-molecule kinetics and super-resolution microscopy by fluorescence imaging of transient binding on DNA origami. *Nano Lett* 2010;10(11):4756-4761.

Kingma, D.P., Ba, Jimmy. Adam: A Method for Stochastic Optimization. In.: arXiv e-prints; 2014. p. arXiv:1412.6980.

Lecun, Y., *et al.* Gradient-based learning applied to document recognition. *P Ieee* 1998;86(11):2278-2324.

Rothemund, P.W.K. Folding DNA to create nanoscale shapes and patterns. *Nature* 2006;440(7082):297-302.

Schlichthaerle, T., *et al.* Site-Specific Labeling of Affimers for DNA-PAINT Microscopy. *Angew Chem Int Ed Engl* 2018;57(34):11060-11063.

Schueder, F., *et al.* An order of magnitude faster DNA-PAINT imaging by optimized sequence design and buffer conditions. *Nat Methods* 2019.
